# Ion-paired antibiotics in PLGA nanoparticles: improving encapsulation efficiency and musculoskeletal infection treatment

**DOI:** 10.1039/d5ra04263a

**Published:** 2025-11-24

**Authors:** Vladislav Frolov, Tamás Sovány, Jan Loskot, Edit Csapó, Norbert Varga, Alharith A. A. Hassan, Ondřej Janďourek, Klára Konečná, Aleš Bezrouk, Eva Šnejdrová

**Affiliations:** a Department of Pharmaceutical Technology, Faculty of Pharmacy in Hradec Králové, Charles University Akademika Heyrovského 1203 500 05 Hradec Králové Czech Republic frolovv@faf.cuni.cz; b Institute of Pharmaceutical Technology and Regulatory Affairs, University of Szeged Eötvös u. 6. H-6720 Szeged Hungary; c Department of Medical Biophysics, Faculty of Medicine in Hradec Králové, Charles University 500 03 Hradec Králové Czech Republic; d Department of Physics, Faculty of Science, University of Hradec Králové Rokitanského 62 500 03 Hradec Králové Czech Republic; e MTA-SZTE Lendület “Momentum” Noble Metal Nanostructures Research Group, University of Szeged Rerrich B. sq. 1 H-6720 Szeged Hungary; f Interdisciplinary Excellence Center, Department of Physical Chemistry and Materials Science, University of Szeged Rerrich B. sq. 1 H-6720 Szeged Hungary; g Department of Biological and Medical Sciences, Faculty of Pharmacy in Hradec Kralove, Charles University Akademika Heyrovského 1203 500 05 Hradec Kralove Czech Republic

## Abstract

Many studies have shown that gentamicin (GEN) and vancomycin (VAN) are effective in the treatment of musculoskeletal infections, especially when applied locally in the form of sustained-release drug delivery systems. A promising strategy in this area appears to be the impregnation of allogeneic bone grafts with antibiotics loaded poly(d,l-lactide-*co*-glycolide) (PLGA) nanoparticles (NPs). However, a major problem in formulating such systems is the high water solubility of these antibiotics, which leads to low drug content in NPs and rapid drug release. In this study, hydrophobic ion pairing (HIP) was employed to enhance the antibiotics loading and their prolong release from PLGA NPs. HIP complexes were formed using three anionic surfactants with bis(2-ethylhexyl) sulfosuccinate sodium salt (AOT) appearing to be the most effective. A novel potentiometric titration method was used to determine the optimal antibiotic-to-surfactant molar ratio. The VAN-AOT and GEN-AOT complexes were encapsulated into NPs prepared with non-commercial PLGA branched on either polyacrylic acid or tripentaerythritol. The size of the optimized nanoparticle formulations was in the range of 160 to 280 nm with the encapsulation efficiency increased to approximately 24% in the case of VAN-AOT and even to 42% in the case of GEN-AOT. The stability of AOT complexes encapsulated in PLGA NPs in aqueous media was investigated using DLS. Subsequently, the microdilution broth method confirmed the antimicrobial efficacy of the free VAN-AOT and GEN-AOT complexes, as well as PLGA NPs loaded with these complexes. Release studies of allogenic bone grafts impregnated with VAN-AOT formulation revealed a three-day release of VAN, whereas GEN-AOT exhibited an almost linear release pattern of GEN, reaching 33% by day 22. These results indicate that bone grafts impregnated with PLGA NPs loaded with HIP-complexed antibiotics represent a promising approach for localized and sustained antibiotic delivery in the treatment of musculoskeletal infections.

## Abstract 2

Many studies have shown that gentamicin (GEN) and vancomycin (VAN) are effective in the treatment of musculoskeletal infections, especially when applied locally in the form of sustained-release drug delivery systems.

## Graphical abstract

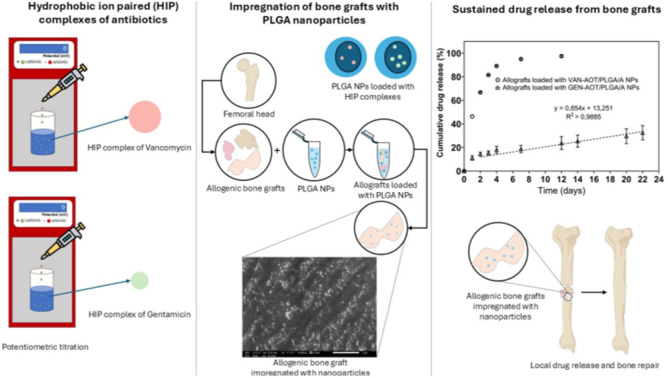

## 1 Introduction

Musculoskeletal infections (MSIs) pose major complications in the field of orthopaedic surgery, leading to high mortality rates, extended hospital stays and poor clinical outcomes. With an aging population, rising rates of fragility fractures, and an increase in joint replacement surgeries, the incidence of MSIs will grow and present a significant socioeconomic challenge.^1^

The standard treatment of bone infections typically involves the removal of necrotic bone and soft tissue, combined with prolonged systemic administration of antibiotics. However, poor vascularization of the bone can hinder the achievement of sufficiently high antibiotic concentrations *via* systemic administration, potentially leading to antimicrobial resistance and systemic side effects.^2,3^ Additionally, pathogens responsible for MSIs often form biofilms, which are difficult to eliminate with systemic antibiotics application.^4,5^ In contrast, combining surgical debridement with local antibiotic delivery has proven to be an effective approach for managing MSIs.^2,6^ After debridement, the resulting dead space should be filled with a biocompatible and biodegradable bone filler that not only ensures optimal local drug concentration, but also promotes bone regeneration.^7,8^

Bone grafts have been proven as a suitable carrier system for local antibiotic applications, showing promise in both the treatment and prevention of infections.^9,10^ At the same time, impregnating a bone scaffold with polymeric particles can modulate the mechanical properties of the scaffold and drug release kinetics.^2,11,12^

Glycopeptides and aminoglycosides have a broad-spectrum activity against Gram-positive and Gram-negative pathogens responsible for MSIs. They have poor tissue penetration when used systemically, but it makes them suitable for local use, as they can't reach the systemic circulation.^4^ Vancomycin (VAN) is a glycopeptide antibiotic that exerts antimicrobial activity against *Staphylococcus aureus*, which is prevalent in bone and joint infections.^13^ Gentamicin (GEN) belongs to the group of aminoglycosides and is commonly used in combination with VAN, which has been proven to be effective in MSIs treatment.^14–16^ Unfortunately, the encapsulation of hydrophilic antibiotics like VAN and GEN into nanoparticles (NPs) based on a hydrophobic poly(d,l-lactide-*co*-glycolide) (PLGA) polymer can be challenging, as the drug tends to partition from the organic phase into the external aqueous phase before the particles solidify.^17–19^ This results in low drug loading and rapid release from the NPs during their preparation.^20^

Drug delivery systems based on the synthetic biopolymer PLGA are approved for clinical use by the Food and Drug Administration (FDA) and the European Medicines Agency (EMA) due to its biocompatibility and biodegradability. Adjusting the molecular weight, monomer ratio, the terminal functional group, and polymer chain architecture of PLGA can change its degradation rate, enabling the customization of drug release profiles to achieve the desired release kinetics.^21,22^ PLGA is the most widely used polymer for localized antibiotic administration and is often employed as a matrix for polymeric NPs loaded with various antimicrobial agents.^23,24^

The previously mentioned challenge of hydrophilic drug partitioning from the polymeric phase can be effectively addressed through the application of hydrophobic ion pairing (HIP). HIP complexation is a method where a charged hydrophilic drug is co-precipitated after forming an electrostatic interaction with an oppositely charged counterion containing hydrophobic moieties. In the case of antibiotics, the HIP preparation involves the ionic bonding of the drug's amino or carboxylic groups with anionic or cationic surfactants, respectively. This approach can modify the solubility of the drug in polar and non-polar solvents, providing sustained release and high drug loading within hydrophobic drug carriers. Furthermore, HIP modification of antibiotics not only preserves but may also increase the antibacterial properties of the drug.^20^

To overcome the limitations associated with the hydrophilic nature of VAN and GEN, the HIP technique was employed to enhance their encapsulation efficiency into PLGA NPs. We prepared HIP complexes of VAN and GEN using bis(2-ethylhexyl) sulfosuccinate sodium salt, sodium dodecyl sulfate, and sodium dodecylbenzene sulfonate as counterions to enhance their encapsulation in PLGA-based NPs and prolong drug release. The molar ratio of the antibiotic–surfactant complexes was determined using a novel potentiometric titration method, and complex formation was confirmed. We investigated how encapsulation efficiency, drug loading, and drug release of prepared NPs were influenced by the type of branching agent used in non-commercial PLGA derivatives, which were branched on polyacrylic acid or tripentaerythritol.

The prepared NPs were used for allogenic bone graft impregnation. The presence of NPs on the surface of allogenic bone grafts was verified, and drug release from NPs-loaded allografts was evaluated, revealing prolonged drug release. We believe that these findings highlight the potential of the developed systems for localized antibiotic delivery in orthopaedic applications.

## 2 Experimental section

### 2.1. Materials

Commercially available drug substances Vancomycin Mylan™ 500 mg inf. plv. sol. as vancomycin hydrochloride (VAN-HCl) equivalent to 500 000 IU and Gentamicini sulfas powder as gentamicin sulfate (GEN-S) were purchase from Dr Kulich Pharma s.r.o (Hradec Kralove, Czech Republic).

Branched PLGA derivatives synthesized originally according to Snejdrova *et al.*^25^ are accurately characterized in Table 1.

**Table 1:** Characteristics of PLGA derivatives for NPs formulation

Polymer designation	LA : GA : br^a^	*M*_w_ (g mol^−1^)	*M*_n_ (g mol^−1^)	End groups	Constitution
PLGA/A	49 : 49 : 2	14 400	8600	Acid	Branched
PLGA/T	48.5 : 48.5 : 3	17 400	5300	Ester	Branched

a LA : GA : br is the ratio of lactic acid (LA), glycolic acid (GA) and branching agent (br) which is either polyacrylic acid (A) or tripentaerythritol (T); *M*_w_ is weight-average molar mass, *M*_n_ is number-average molar mass.

In brief, branched copolymers were synthesized by hot-melt condensation polymerization of an equimolar mixture of glycolic acid and dl-lactic acid in the presence of 2% polyacrylic acid (A) or 3% tripentaerythritol (T) as branching agents. All reagents were obtained from Merck KGaA (Darmstadt, Germany). The polymerization was conducted at 160 °C under a pressure of 550 Pa for 90 hours.

Poloxamer 407 (Pluronic-F127), ethyl acetate (EtAc), dimethyl sulfoxide (DMSO), methanol, phosphate-buffered saline (PBS) of pH 7.4, *o*-phtaldialdehyde (OPA) and anionic surfactants bis(2-ethylhexyl) sulfosuccinate sodium salt (AOT); sodium dodecyl sulfate (SDS); sodium dodecylbenzene sulfonate (SDBS), used as ion pairing reagents, were obtained from Merck KGaA (Darmstadt, Germany). 2-mercaptoethanol, isopropanol, trifluoroacetic acid (TFA) (HPLC grade) and acetonitrile (HPLC grade) were purchased from Thermo Fisher Scientific Co., Ltd (Waltham, MA, USA).

The allogeneic femoral head, which was morselised into small pieces with an approximate size of 1–3 mm, was obtained from a deceased human donor and provided to the authors for scientific purposes by the Tissue Bank of University Hospital of Hradec Kralove, Czech Republic.

### 2.2. Hydrophobic ion-pairing of vancomycin and gentamicin

Three anionic surfactants were investigated as counterions: AOT, SDS and SDBS. Molar ratio of counterion to antibiotic, which corresponds to zero complex charge, was determined *via* titration using a Mütek PCD 02 particle charge detector (Mutek, Herrsching, Germany), as described previously.^26^ Potentiometric titration was employed to determine the neutral charge of both the VAN or GEN complexes with hydrophobic ion pairs (VAN-HIP or GEN-HIP, respectively). To find the neutral charge of the VAN-HIP complex, 10 mL of VAN-HCl solution (1 mg mL^−1^) in 0.01 M HCl (pH 2) was added into the measure cell and titrated with aqueous solutions of counterions: AOT, SDS or SDBS (2 mg mL^−1^). For the GEN-HIP complex, 10 mL of GEN-S (0.5 mg mL^−1^) in a buffered aqueous medium (10 mM sodium acetate, 10 mM KCl, 10 mM CaCl_2_, pH 5) was titrated with the same aqueous solutions of counterions at a concentration of 5 mg mL^−1^.

VAN-HIP was prepared using the organic solvent-free method described by Efiana *et al.*^27^ In brief, a solution containing VAN-HCl in 0.01 M HCl was prepared. The aqueous solution of counterion (AOT, SDS or SDBS) was added dropwise in a predetermined molar ratio.

The mixing of antibiotic and counterion solutions was carried out using a magnetic stirrer at 500 rpm for 2 h.

GEN-HIP was prepared as described previously by Ter Boo *et al.*^28^ with slight modifications. GEN complexes were formed using buffer solution (10 mM sodium acetate, 10 mM KCl, 10 mM CaCl_2_, pH 5) of GEN-S (0.5 mg mL^−1^) and aqueous solutions of counterions: AOT, SDS and SDBS (5 mg mL^−1^). The GEN-HIP was then formed by following the same procedure described above for the VAN-HIP.

HIP complexes of VAN and GEN were separated from the supernatant by centrifugation at 10 000 rpm for 10 min at room temperature, washed with 0.01 M HCl solution, and then freeze-dried (FreeZone Triad, Labconco, Kansas, USA) and stored at −20 °C until further use.

### 2.3. Fourier transform infrared spectroscopy (FTIR) of ion-paired complexes

The infrared spectra of VAN-HIP, GEN-HIP, VAN-HCl, GEN-S, and the counterions AOT, SDS, and SDBS were obtained by an Avatar 330 FT-IR apparatus (Thermo Fisher Scientific Inc., Waltham, MA, USA) coupled with Zn/Se horizontal attenuated total reflectance (HATR) equipment. The spectra were collected in the range from 400 to 4000 cm^−1^, and 128 scans were used for each spectrum. The graphical evaluation of the acquired spectra was performed using SpectraGryph (version 1.2.15.; Dr Friedrich Menges Software, Entwicklung, Obersdorf, Germany).

### 2.4. Analysis of VAN-HCl and its ion-paired complex with AOT

The amount of VAN-HCl and its hydrophobic ion pair was determined by HPLC (Agilent 1200; Agilent Technologies, Santa Clara, USA), using a modified version of a protocol described previously by Efiana *et al.*^27^ Restek Ultra 100A column C18 with dimensions 150 mm × 4.6 mm, 5 µm (Restek, PA, USA) was maintained at 25 °C. Chromatography was performed using the isocratic elution method with the mobile phase 0.01% (v/v) TFA in Milli-Q water and acetonitrile at a volume ratio of 90 : 10. The flow rate was set to 1.0 mL min^−1^, and the injection volume was 20 µL. UV detection was set at 214 nm. Retention time of both VAN-HCl and VAN-HIP was approximately 6 min and the total run time 10 min.

### 2.5. Analysis of GEN-S and its ion-paired complex with AOT

The amount of GEN-S and GEN-AOT was determined using OPA assay, as described by Zhang *et al.*,^29^ with minor modifications. The OPA reagent was prepared by adding 0.5 g of *o*-phtaldialdehyde in 12.5 mL of methanol and 0.6 mL of 2-mercaptoethanol. To this solution, 112.5 mL of 0.4 M sodium borate buffer (pH 9.7) was added. 1 mL of the GEN-S or GEN-AOT sample, 1 mL of isopropanol, and 1 mL of OPA were mixed and incubated in the dark at room temperature for 30 min. Absorbance was measured at 332 nm using Specord 250 Plus (Jena Analytik, Jena, Germany).

### 2.6. Precipitation efficiency of ion-paired complexes

The precipitation efficiency of the HIP complex represents the percentage of the drug incorporated into the solid precipitate relative to the total amount of drug initially used for complex formation. The precipitation efficiency of the antibiotics complexed with ion-pairing agents at ratios determined from potentiometric titration was evaluated by quantifying the residual free drug in the supernatant using HPLC and UV spectroscopy. Each sample was prepared three times and analyzed. Precipitation efficiency was calculated as follows:

Precipitation efficiency (%) = 100 − (*C*_s_/*C*_t_ × 100), where *C*_s_ is antibiotic concentration in supernatant and *C*_t_ is the total antibiotic concentration.

### 2.7. Preparation of PLGA NPs loaded with GEN ion-paired complex with AOT

GEN-AOT-loaded PLGA NPs were prepared by a single emulsion-solvent evaporation technique using EtAc as an organic solvent. First, 5 mg of GEN-AOT complex and 100 mg of PLGA were dissolved in 1 mL EtAc. Aqueous 0.5% (w/v) Pluronic-F127 solution (5 mL) was added to the organic phase and sonicated on ice for 30 s with a power of 40 W using a Sonopuls HD 2070 (Bandelin electronic GmbH & Co, Berlin, Germany) to form an oil-in-water (O/W) emulsion. The resulting O/W emulsion was continuously stirred at room temperature for 1.5 h to evaporate EtAc. The NPs were gradually isolated by centrifugation (8000 rpm, 10 min and 15 000 rpm, 15 min, 4 °C) and washed with PBS of pH 7.4 to remove the complexes which were not encapsulated.

### 2.8. Preparation of PLGA NPs loaded with VAN ion-paired complex with AOT

VAN-AOT-loaded PLGA NPs were prepared by the nanoprecipitation method. 5 mg of VAN-AOT complex and 100 mg of PLGA were dissolved in DMSO (1 mL) and added dropwise to aqueous 0.5% (w/v) Pluronic-F127 solution (5 mL) with moderate stirring at room temperature.

The NPs were isolated by centrifugation (8000 rpm, 10 min and 15 000 rpm, 15 min, 4 °C) and washed with PBS of pH 7.4 to remove the complexes which were not encapsulated.

### 2.9. Measurement of NPs size and polydispersity

The size and polydispersity index (PDI) of PLGA NPs loaded with HIP complexes were analyzed by DSL using a Zetasizer Nano ZS (Malvern Panalytical, Malvern, UK) equipped with a red helium-neon laser operating at a wavelength of 632.8 nm. All measurements were performed at a temperature of 25 °C and a backscattering angle of 173°. Prior to analysis, each sample was diluted 20-fold with purified water. A volume of 1 mL of the diluted sample was then transferred into a disposable polystyrene cuvette.

The stability of PLGA NPs loaded with AOT complexes in aqueous media was evaluated using DLS, as previously described by Lu *et al.*^30^ PLGA NPs were diluted tenfold with Milli-Q water or PBS (pH 7.4; ionic strength 163 mM) and maintained at room temperature. Their stability was monitored by DLS measurements at 0, 3, 24, and 72 hours. All measurements were conducted in triplicates.

### 2.10. Determination of drug loading and encapsulation efficiency

A 0.5 mL aliquot of the nanosuspension was centrifuged and washed. The VAN-AOT-loaded NPs were then dispersed in 1 mL of acetonitrile, and 2 mL of methanol was added to precipitate the PLGA. The suspension was centrifuged at 15 000 rpm for 10 minutes at room temperature, and the supernatant was analyzed for VAN (see Section 2.3). The GEN-AOT-loaded NPs were hydrolyzed in a 0.1 M NaOH solution, and after centrifugation, the supernatant was diluted with a borate buffer at pH 9.7. Each sample was analyzed for GEN (see Section 2.4). All measurements were performed in triplicates.

The encapsulation efficiency (EE%) of the VAN-AOT and GEN-AOT complexes in PLGA NPs was calculated as the percentage of the mass of the complex encapsulated within the nanoparticles relative to the initial mass of the AOT complex used for NPs preparation. The drug loading (DL%) was defined as the percentage of the encapsulated AOT complex mass relative to the total mass of the obtained PLGA NPs (polymer and complex). EE% represents the effectiveness of the encapsulation process, while DL% indicates the amount of complex incorporated per unit mass of NPs. Both parameters are interrelated, as they depend on the mass of encapsulated complex; however, their denominators differ. EE% is based on the initial amount of complex used in the formulation, whereas DL% is calculated with respect to the total mass of the NPs. Consequently, an increase in the drug input may enhance DL% only if EE% remains sufficiently high.

DL% and EE% were determined as:DL (%) = (mass of AOT − complex loaded/total mass of NPs) × 100EE (%) = (mass of AOT − complex loaded/initial mass of AOT − complex) × 100

### 2.11. Scanning electron microscopy of PLGA nanoparticles

The morphology of the PLGA NPs loaded with VAN-AOT and GEN-AOT complexes was observed with a field-emission scanning electron microscope JSM-IT500HR (JEOL, Tokyo, Japan). The SEM was operated in secondary electrons (SE) mode at an accelerating voltage of 15 kV. To mitigate charging effects and improve image quality, all samples were coated with a 6 nm thick layer of gold using an EM ACE200 sputter coater (Leica Microsystems, Wetzlar, Germany).

### 2.12. Scanning electron microscopy and energy dispersive X-ray spectroscopy of bone grafts

The surface morphology of allogenic bone grafts was examined using a scanning electron microscope FlexSEM 1000 (Hitachi, Tokyo, Japan). SEM observations were made in a secondary electrons (SE) mode and a backscattered electrons (BSE) mode at an accelerating voltage of 15 kV. Additional images with a higher resolution were taken with a field-emission scanning electron microscope JSM 7500F (JEOL, Tokyo, Japan) working in SE mode at an accelerating voltage of 15 kV.

Allogenic bone grafts, each sample weighing 1 g, were impregnated by mixing for 30 min with (a) 100 mg of VAN-HCl powder, (b) 100 mg of GEN-S powder, (c) 3 mL of PLGA NPs suspension loaded with the VAN-AOT complex or (d) 3 mL of PLGA NPs suspension loaded with the GEN-AOT complex. Blank samples of allografts and bone grafts impregnated with antibiotic powders were fixed in 3% (v/v) glutaraldehyde (EMS Inc., Hatfield, PA, USA) diluted in PBS. Then they were dehydrated in an ethanol series (concentrations 50, 80, 90, 95, and 99% (v/v), 20 min per each step) and dried in an EM CPD 300 critical point drying device (Leica Microsystems, Wetzlar, Germany). Samples impregnated with PLGA NPs were prepared by drying at room temperature. To mitigate charging effects and improve image quality, all samples were coated with a 6 nm thick layer of gold using an EM ACE200 sputter coater (Leica Microsystems, Wetzlar, Germany).

To determine the drug distribution on the impregnated bone grafts, SEM imaging was supplemented with elemental mapping using the energy-dispersive X-ray spectroscopy (EDS) technique. For this purpose, both blank bone grafts and those impregnated with antibiotic powders or PLGA NPs containing AOT complexes were dried at room temperature and coated with a 6 nm thick copper layer. Coating with copper was more suitable for the EDS analysis than coating with gold, since a strong peak of Au overlaps with the characteristic peaks of P and S in EDS spectra. Sulfur was used to indicate the presence of the drug (VAN-AOT or GEN-AOT) on the bone graft surface.

### 2.13. *In vitro* antibacterial activity screening

The microdilution broth method was performed according to EUCAST (The European Committee on Antimicrobial Susceptibility Testing) instructions^31^ with slight modifications. Two testing bacterial strains (G+ and G−) were purchased from the Czech Collection of Microorganisms (CCM, Brno, Czech Republic): methicillin-resistant *Staphylococcus aureus* subsp. *aureus* (MRSA) CCM 4750 (ATCC 43300) and *Pseudomonas aeruginosa* CCM 3955 (ATCC 27853). The cultivation was performed in cation-adjusted Mueller–Hinton broth (CAMHB, M–H 2 Broth, Merck, Darmstadt, Germany) at 35 ± 2 °C. Tested compounds were dissolved in DMSO (Merck) or in water for injection (Ardeapharma, Sevetin, Czech Republic) to produce stock solutions. In cases where DMSO was used, the final concentration of DMSO in the cultivation medium did not exceed 1% (v/v) of the total solution composition and did not affect the growth of bacteria. Positive growth controls consisted of test microbe solely, negative growth controls consisted of cultivation medium. Antibacterial activity was expressed as minimum inhibitory concentration (MIC, in µg mL^−1^) and was read after 24 and 48 h of static incubation in the dark and humidified atmosphere, at 35 ± 2 °C. Visual inspection was used for MIC endpoint evaluation that was indicated by inhibition of bacterial growth. All compounds were tested in tetraplicates.

### 2.14. Drug release studies

The *in vitro* dissolution of HIP complexes from the PLGA NPs as well as *ex vivo* drug release from allografts impregnated with PLGA NPs was performed using the dialysis bag method. Briefly, 3 mL of NPs suspension was centrifuged, washed, and redispersed in 3 mL of dissolution medium (PBS, pH 7.4), added to a dialysis bag, and placed into 6 mL of PBS of pH 7.4. Bone grafts were impregnated with NPs as described before.^32^ 1 g of allografts were impregnated for 30 min with 3 mL of washed NPs suspension and subsequently added to the dialysis bag with 3 mL of dissolution medium and placed into 6 mL of PBS of pH 7.4. Samples were incubated under dynamic conditions at 37 °C and 80 rpm in a Julabo SW 22 shaking water bath (Labortechnik GmbH., Seelbach, Germany). At the predetermined time points (2 h, 5 h and subsequently on days 1, 2, 3, 4, 7, 12, 14, 20 and 22) the release medium was collected and replaced with 6 mL of fresh PBS. The amount of antibiotic released into the dissolution medium was determined as described in Sections 2.3 and 2.4.

## 3. Results and discussion

Bone grafts impregnated with antibiotic-loaded PLGA nanoparticles represent a biocompatible and biodegradable bone filler that not only provides therapeutically effective local drug concentration in case of musculoskeletal infection and prolonged antibiotic administration but also promotes bone regeneration. The problem of low loading of hydrophilic antibiotics vancomycin and gentamicin, which are effective in MSIs therapy, into hydrophobic PLGA polymer was solved in our study by hydrophobic ion pairing of antibiotics with anionic surfactants as well as by choosing a PLGA derivative with lower hydrophobicity.

Two polyester derivatives of PLGA were previously synthesized *via* catalyst-free direct melt polycondensation of glycolic acid and dl-lactic acid using tripentaerythritol or polyacrylic acid as branching monomers, yielding branched PLGA structures.^25^ As the reaction was carried out without a catalyst, purification steps such as dissolution, precipitation, and drying were not required. The synthesis process is straightforward, well-controlled, and readily scalable. The resulting derivatives exhibited low molar mass and a branched configuration. These branched PLGA derivatives were subsequently employed for NPs formulation in this study.

### 3.1. Preparation of hydrophobic ion-paired complex

**Fig. 1:**
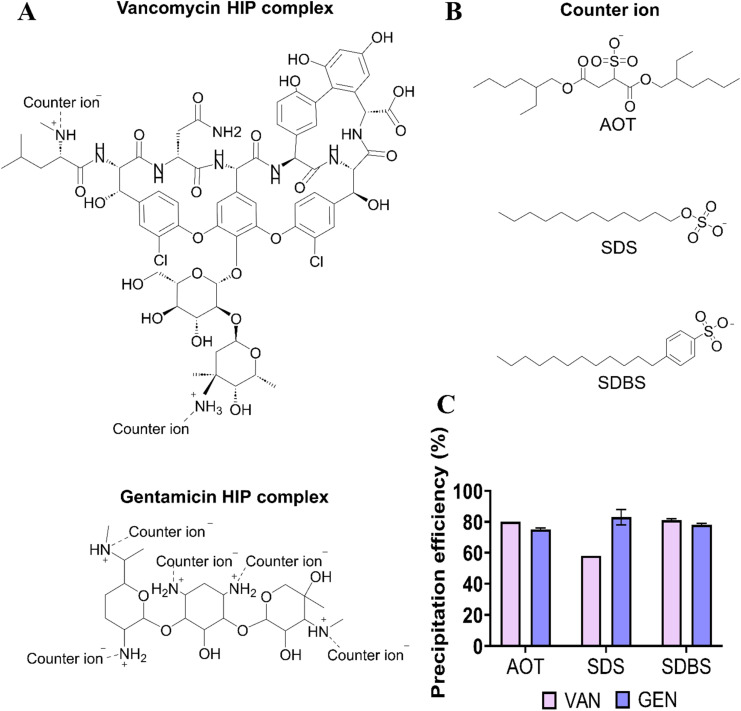
The scheme of antibiotics hydrophobic ion-paired (HIP) complexes (A) and – chemical structure of surfactants bis(2-ethylhexyl) sulfosuccinate sodium salt (AOT), sodium dodecyl sulfate (SDS) and sodium dodecylbenzene sulfonate (SDBS) used as counter ions (B), precipitation efficiency of vancomycin (VAN) and gentamicin (GEN) ion-paired with anionic surfactants (C). The results are expressed as mean ± SD of three measurements.

An organic solvent-free approach was utilized for the formation of both VAN and GEN HIP complexes. Prior to the preparation of the HIP complexes, the optimal surfactant to antibiotic molar ratio required to reach a neutral complex charge was determined using a novel potentiometric titration method.^26^ It is crucial that the surfactant remained soluble in the acidic aqueous solution without precipitating out of the system. Accordingly, the anionic surfactants AOT, SDS and SDBS were selected as counterions due to their low p*K*_a_ values of −0.75, −1.5, and −1.87, respectively, which enabled them to maintain their negative charge in the acidic aqueous environment.^33^ VAN possesses two positively charged amino groups, while GEN has five (Fig. 1A), which can interact with the negatively charged sulfonate groups of the counterions containing hydrophobic domains such as alkyl tails and an aromatic ring (Fig. 1B), allowing the entire complex to be hydrophobic. This resulted in a higher amount of surfactant being required to achieve a neutral charge for the GEN complex compared with the VAN complex (Table 2).

**Table 2:** Amounts of counter ion (mol) required to reach zero complex charge^a^

Counter ion	Amount of counter ion (mol) mean ± SD	
	VAN	GEN
AOT	2.2 ± 0.1	3.9 ± 0.1
SDS	2.3 ± 0.1	4.6 ± 0.2
SDBS	2.4 ± 0.1	4.9 ± 0.2

a VAN – vancomycin, GEN – gentamicin, AOT – bis(2-ethylhexyl) sulfosuccinate sodium salt, SDS – sodium dodecyl sulfate, SDBS – sodium dodecylbenzene sulfonate.

### 3.2. Precipitation efficiency of hydrophobic ion-paired complex

The preparation of GEN-HIP complexes using the determined molar ratios demonstrated precipitation efficiencies of up to 75% or higher for all surfactants (Fig. 1C).

For VAN, precipitation efficiencies using AOT and SDBS as counterions reached around 80%, while SDS achieved approximately 58%. These results suggest the effectiveness of the novel potentiometric method in identifying the surfactant-to-drug ratio required to achieve sufficient antibiotic precipitation. However, 100% precipitation efficiency was not observed for any of the ratios used, indicating that a higher counterion concentration may be necessary to reach complete precipitation than the amount required to form a neutral complex. Prior studies have reported that a 100% precipitation efficiency of VAN was achieved by using a surfactant-to-drug molar ratio 3 : 1, which was higher than the expected 2 : 1 ratio.^27^

Similar findings were reported by Wibel *et al.*, who observed that the highest precipitation efficiency between VAN and the counterion was attained at a charge ratio of 2.5.^34^ This can be attributed to the fact that for complete drug precipitation, not only electrostatic interactions but also hydrophobic interactions are crucial between the hydrophobic moieties of the counterion.^35^ Simultaneously, the selected ratios were utilized in subsequent preparations to minimize side effects associated with excessive surfactant concentrations in the complex while ensuring cost-effectiveness.^36^

### 3.3. Fourier transform infrared spectroscopy (FTIR) of ion-paired complexes

**Fig. 2:**
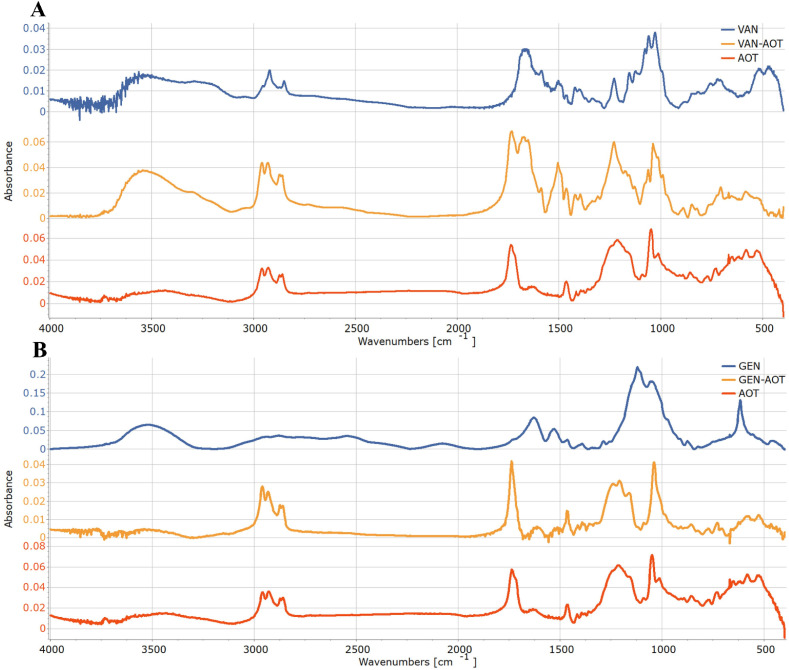
FTIR spectra of vancomycin (VAN) (A) and gentamicin (GEN) (B) complexes with bis(2-ethylhexyl) sulfosuccinate sodium salt (AOT). AOT (red line), vancomycin or gentamicin (blue line), and the respective complex with AOT (orange line).

Previously, FTIR analysis was performed by other investigators to confirm the interaction between amino groups of antibiotics and the sulfate group of the anionic surfactants.^27,37^Fig. 2 illustrates infrared spectra of GEN-S, VAN-HCl, AOT, and their complexes prepared using the ratios found by the electrostatic titration method.

VAN has a broad peak from 3200 to 3700 cm^−1^ that corresponds to OH stretching of alcoholic groups and carboxylic acid at the C terminus of the peptide. The stretching of the NH and NH_2_ groups gives only a very weak signal at 3039 cm^−1^. The peaks at 2954 cm^−1^ and 2922 cm^−1^ correspond with the asymmetric and symmetric stretching of the CH_3_, respectively, while at 2872 cm^−1^ and 2850 cm^−1^ to the asymmetric and symmetric stretching of the CH_2_ groups, respectively. The wide high intensity peak at 1666 cm^−1^ corresponds to C=O stretching of the amide bands, while the one at 1588 cm^−1^ to the stretching of the C–N–H part of the amide band.

The signals of bending and rocking of NH groups can be found at 1503 cm^−1^ and 1155 cm^−1^, respectively. The high intensity peaks at 1060 cm^−1^ and 1029 cm^−1^ corresponds with the C–O stretching within the VAN molecule. The shift of the NH rocking from 1155 cm^−1^ to 1176 cm^−1^ in the VAN-AOT spectrum might indicate the complexation.

The AOT has characteristic peaks at 3435 cm^−1^, that corresponds to the OH stretching in its H_2_O content, at 2960 cm^−1^, 2929 cm^−1^, that corresponds to asymmetric, and at 2873 cm^−1^ and 2860 cm^−1^, that corresponds to symmetric stretching of CH_3_ and CH_2_ groups, respectively. The high intensity peak at 1737 cm^−1^ corresponds to the presence of carbonyl groups, the medium intensity peak at 1466 cm^−1^ is the bending peak of CH_2_ groups. The high intensity peaks at 1215 cm^−1^, and 1050 cm^−1^ corresponds with the asymmetric and symmetric stretching of SO_3_^−^ groups while the peaks at 1014 cm^−1^ and 858 cm^−1^, corresponds to the asymmetric and symmetric stretching of the S–O–C band. The right shift of the asymmetric and symmetric SO_3_ stretching to 1176 cm^−1^ and 1039 cm^−1^ in the VAN-AOT spectrum supports the successful formation of HIP complex. Similar shifts can also be observed in case of VAN-SDS (Fig. S1A) and VAN-SDBS (Fig. S2A) complexes where the peaks of SO_3_ stretching have shifted from 1191 cm^−1^ to 1172 cm^−1^ and from 1046 cm^−1^ to 1033 cm^−1^, and from 1084 cm^−1^ to 1061 cm^−1^ respectively.

GEN has a broad peak of OH stretching 3520 cm^−1^ while the wide peak of NH stretching may be found at around 3100 cm^−1^, but it overlaps with the asymmetric stretching of CH_2_ at 2938 cm^−1^. The symmetric CH_2_ stretching can be found at 2880 cm^−1^. The most distinctive peak of the spectrum is the bending of the primary amine at 1630 cm^−1^ as none of the tested surfactants have it.^28,38^ Other characteristic peaks are the CH_2_ bending at 1466 cm^−1^ and the OH bending at 1393 cm^−1^. The high intensity peaks at 1122 cm^−1^ and 1056 cm^−1^ corresponds to C–O stretching. The right shift of the bending of the amine band from 1630 cm^−1^ to 1612 cm^−1^ in the GEN spectrum and the shift of the symmetric SO_3_ stretching from 1050 cm^−1^ to 1040 cm^−1^ in the AOT spectrum supports the successful formation of the HIP complex. Similar shifts can also be observed in the spectra of the GEN-SDS (Fig. S1B) and GEN-SDBS (Fig. S2B) complexes too.

### 3.4. NPs preparation and characterization

The double emulsion solvent evaporation approach is a widely utilized technique for the fabrication of PLGA-based NPs loaded with hydrophilic drugs.^39^ Unfortunately, PLGA-based particulate systems often struggle to achieve high encapsulation efficiency for hydrophilic drugs due to the hydrophobicity of the PLGA. The encapsulation of hydrophilic and amphiphilic molecules is challenging, as the drug tends to migrate from the organic phase into the external aqueous phase before the particles solidify.^19,40,41^

As demonstrated in published studies, the incorporation of more lipophilic drugs into PLGA NPs does not experience external leakage into the aqueous phase, leading to enhanced EE.^42^ After HIP, drug hydrophilicity was reduced and techniques typically employed for the encapsulation of hydrophobic molecules, such as single emulsion-solvent evaporation and nanoprecipitation, became applicable for the GEN-AOT and VAN-AOT complexes, respectively. Despite the use of HIP, VAN retained its hydrophilic properties due to the inadequate coverage of the molecule by the hydrophobic groups of the surfactant. This was evidenced by the poor solubility of the complexes in organic solvents commonly utilized for NPs preparation, such as dichloromethane, acetone, or ethyl acetate.^39^ Consequently, the only organic solvent capable of simultaneously dissolving the PLGA and VAN-AOT complex was DMSO, which could then be employed in the nanoprecipitation technique owing to its miscibility with water. To the best of the author's knowledge, this study represents the first reported application of HIP to encapsulate VAN within PLGA-based polymeric NPs.

**Fig. 3:**
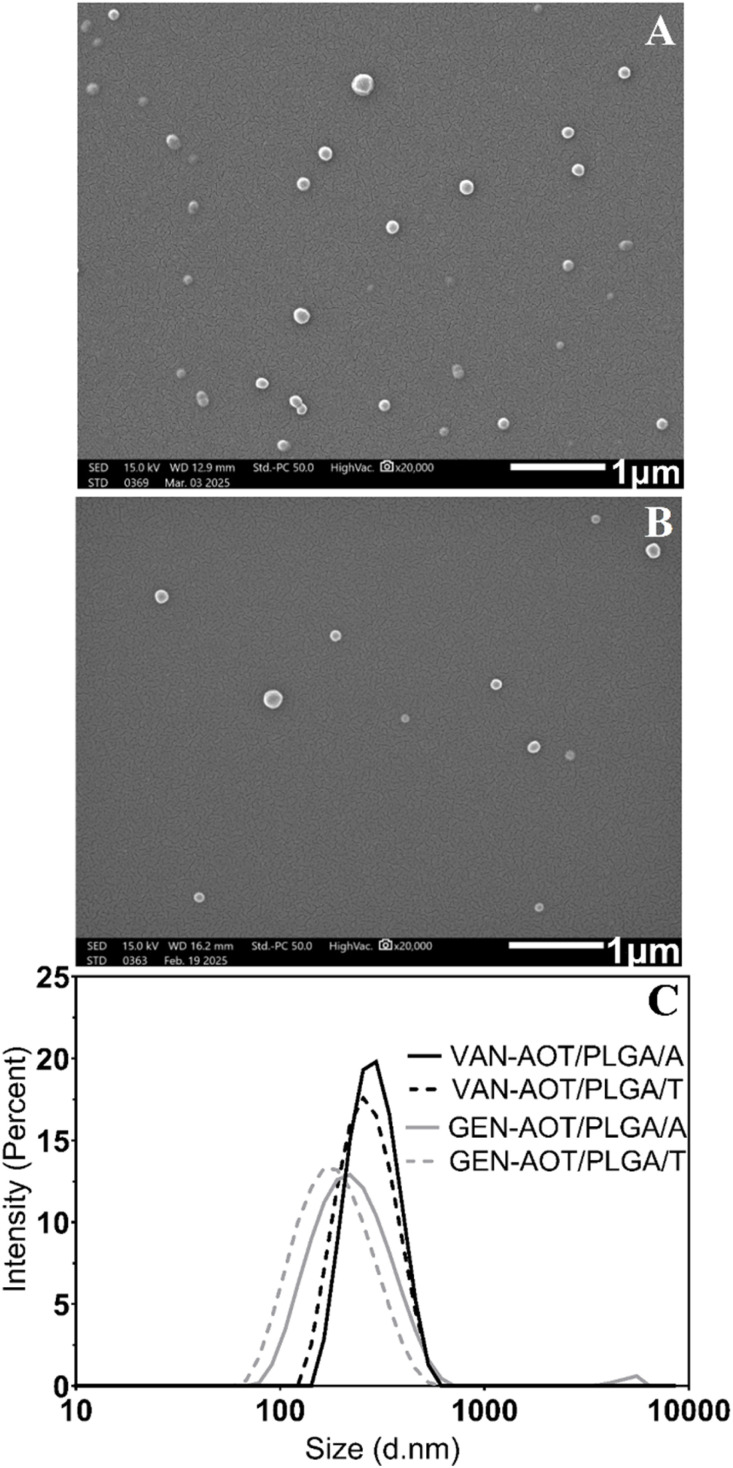
SEM images of GEN-AOT-loaded (A) and VAN-AOT-loaded (B) PLGA/A nanoparticles. DLS size distribution curves (C) of VAN-AOT-loaded PLGA/A nanoparticles (black line), VAN-AOT-loaded PLGA/T nanoparticles (black dashed line), GEN-AOT-loaded PLGA/A nanoparticles (grey line), and GEN-AOT-loaded PLGA/T nanoparticles (grey dashed line).

VAN-AOT and GEN-AOT were selected as drug complexes for the subsequent preparation of PLGA NPs. SEM observations of PLGA NPs have shown that both the NPs loaded with GEN-AOT (Fig. 3A) and those loaded with VAN-AOT (Fig. 3B) have a smooth surface and spherical shape.

The size distribution and mean particle size of the PLGA NPs were characterized by dynamic light scattering (DLS) measurements. A narrow size distribution was observed across all the prepared PLGA NPs formulations (Fig. 3C).

As shown in Table 3, the largest particles were observed in the VAN-AOT/PLGA/A formulation, exhibiting an average diameter of 281 ± 10 nm, while the smallest particles were found in the GEN-AOT/PLGA/T formulation, with an average diameter of 167 ± 3 nm. In both preparation methods, the mean particle size and polydispersity index (PDI) varied depending on the type of PLGA derivative used. Specifically, NPs formulated with PLGA/A exhibited larger particle sizes compared to those prepared with PLGA/T, when using the same method.

**Table 3:** Mean diameter, polydispersity index (PDI), encapsulation efficiency (EE) and drug loading (DL) of VAN-AOT-loaded and GEN-AOT-loaded PLGA NPs. The results are expressed as mean ± SD of three measurements

Formulation	Mean diameter (nm)	PDI (−)	EE (%)	DL (%)
VAN-AOT/PLGA/A	281 ± 10	0.07 ± 0.02	23.8 ± 2.1	1.5 ± 0.2
GEN-AOT/PLGA/A	209 ± 1	0.20 ± 0.01	42.1 ± 0.8	2.9 ± 0.1
VAN-AOT/PLGA/T	254 ± 2	0.10 ± 0.03	12.4 ± 0.8	0.8 ± 0.1
GEN-AOT/PLGA/T	167 ± 3	0.15 ± 0.01	16.4 ± 3.3	1.5 ± 0.2

### 3.5. Stability of PLGA nanoparticles loaded with hydrophobic-ion paired complexes with AOT

The stability of VAN-AOT and GEN-AOT loaded PLGA/A NPs was evaluated by monitoring their size distribution following a 10-fold dilution in Milli-Q water or PBS at room temperature.

All PLGA/A NPs loaded with VAN-AOT and GEN-AOT complexes demonstrated excellent stability in Milli-Q water, showing no significant changes in hydrodynamic size or PDI over a period of 72 hours (Fig. S3 and S4). In contrast, VAN-AOT-loaded PLGA/A NPs exhibited gradual size reduction over time upon dilution in PBS. This behavior indicates progressive degradation of the PLGA matrix and partial dissolution of the encapsulated antibiotic. These size changes were accompanied by an increase in PDI, reflecting the formation of a more heterogeneous population of NPs.

This instability can be attributed to insufficient counterion coverage of the VAN molecule. The limited number of available amino groups (two per molecule; Fig. 1) restricts the formation of strong ion-pair interactions with the counterion, thereby facilitating ion exchange with PBS ions and promoting complex dissociation and drug release.

In contrast, GEN, being a smaller molecule with a higher molar ratio of counterion, forms a more stable HIP complex that provides enhanced electrostatic shielding. Consequently, GEN-AOT-loaded PLGA/A NPs maintained their original size and PDI in PBS for up to 72 hours.

### 3.6. Drug loading and encapsulation efficiency

The EE and DL of the prepared PLGA NPs were determined using a direct method. This involved measuring the amount of loaded HIP-complex after dissolution in an organic solvent (VAN-AOT) or hydrolysis (GEN-AOT) of the PLGA-based NPs. When PLGA/A was used for NPs preparation, the observed EE and DL of NPs with VAN-AOT and GEN-AOT complexes were approximately two times higher compared with NPs prepared with PLGA/T (Table 3).

The observed difference in EE and DL between the PLGA/A-based and PLGA/T-based NPs can be attributed to the stronger binding affinity of the antibiotic complexes to the PLGA/A polymer. This polymer contains a negatively charged terminal carboxylic acid group, which may facilitate the formation of hydrogen bonds with the hydrophilic groups of antibiotics that are not occupied by the counterions.

### 3.7. Scanning electron microscopy and energy dispersive X-ray spectroscopy of bone grafts

**Fig. 4:**
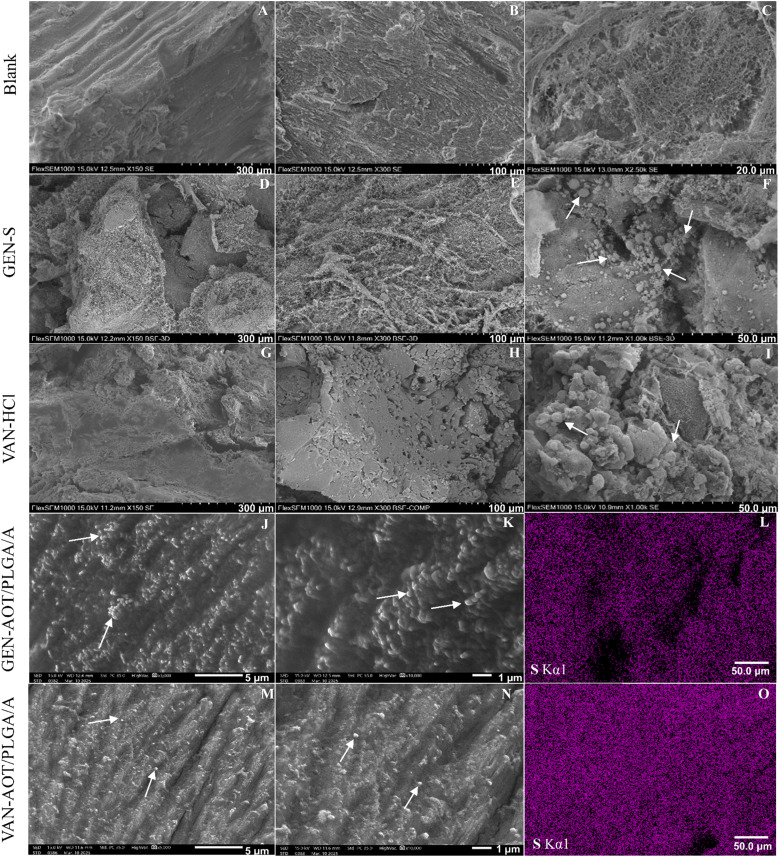
SEM images of blank allogenic bone grafts (A–C), grafts loaded with the GEN-S substance (D–F), with the VAN-HCl substance (G–I), GEN-AOT/PLGA/A (J and K) or VAN-AOT/PLGA/A (M and N) NPs. The particles of antibiotic powders and PLGA/A NPs are indicated with arrows. EDS maps of sulfur distribution on the grafts loaded with GEN-AOT/PLGA/A (L) or VAN-AOT/PLGA/A NPs (O).

SEM imaging of the allogenic bone grafts was conducted to see their surface morphology and verify the presence of GEN-S and VAN-HCl powders and PLGA NPs on their surface. The bone graft morphology is generally heterogenous, showing the presence of soft tissues (Fig. 4A), rough, plate-like bone structures (Fig. 4B) or porous bone structures (Fig. 4C). Blank bone grafts (Fig. 4A–C) differ visually from the bone grafts loaded with GEN-S (Fig. 4D–F) and VAN-HCl (Fig. 4G–I) powders. The particles of GEN-S (Fig. 4F) and VAN-HCl (Fig. 4I) powders were observed on the surface of bone grafts loaded with these substances.

The presence of PLGA NPs was confirmed on the surface of the respective bone graft after its impregnation (Fig. 4J, K, M and N).

EDS maps have shown a homogeneous distribution of sulfur, and thus also the drug, on the bone grafts loaded with GEN-AOT/PLGA/A NPs (Fig. 4L), as well as on those loaded with VAN-AOT/PLGA/A NPs (Fig. 4O). Sulfur could be used as an indicator of the drugs, because it is contained in the VAN-AOT and GEN-AOT HIP complexes, but it was below the detection limit in blank bone graft samples. EDS maps of other chemical elements detectable in the drug-loaded and blank samples are provided in the SI (Fig. S5–S9).

### 3.8. *In vitro* antibacterial activity screening

The antibacterial activity was screened for VAN-HCl and GEN-S, their ion-paired complexes with AOT, PLGA NPs loaded with these complexes, AOT surfactant, and empty PLGA NPs, against clinically relevant bacterial strains mainly associated with MSIs, namely methicillin-resistant *Staphylococcus aureus* (MRSA) and *Pseudomonas aeruginosa* (PA). Results are presented in Table 4.

**Table 4:** *In vitro* antibacterial activity of vancomycin hydrochloride (VAN-HCl), gentamicin sulfate (GEN-S), ion-paired complexes of vancomycin and gentamicin with bis(2-ethylhexyl) sulfosuccinate sodium salt (AOT) and PLGA nanoparticles loaded with complexes against two bacterial strains. Results are expressed as minimum inhibitory concentrations (MICs) in µg mL^−1^

Bacterial strain	MIC (µg ml^−1^)			
	*Staphylococcus aureus* subsp. *aureus* methicilin-resistant (MRSA) (CCM 4750, ATCC 43300)		*Pseudomonas aeruginosa* (PA) (CCM 3955, ATCC 27853)	
	24 h	48 h	24 h	48 h
VAN-HCl	2	2	>64	>64
GEN-S	>64	>64	1	1–2
VAN-AOT	1–2	2	>64	>64
GEN-AOT	>64	>64	0.5	0.5–1
VAN-AOT/PLGA/A	0.5	0.5	>32	>32
GEN-AOT/PLGA/A	>64	>64	0.5–1	1–2

Antibacterial activity of VAN-HCl and GEN-S showed typical action against both strains based on their spectrum. VAN-HCl, which is usually used against G+ bacteria, inhibited growth of MRSA at concentration 2 µg mL^−1^. GEN-S, usually effective against G− microbes, inhibited growth of PA at concentration 1–2 µg mL^−1^.

The activity of the VAN-AOT and GEN-AOT was comparable showing that HIP complexation did not negatively affect the antimicrobial activity. The activity of AOT itself in the same concentrations corresponding to the amount of AOT in complexes was negligible showing no growth inhibition. PLGA NPs loaded with VAN-AOT or GEN-AOT proved to have the same activity as VAN-HCl and GEN-S or slightly improved in case of VAN-AOT/PLGA/A NPs. Suspensions of PLGA without HIP complexes did not inhibit the growth of included bacterial strains. It can be concluded that HIP complexation and subsequent encapsulation into PLGA NPs produced comparable or improved results without negatively affecting the activity of VAN and GEN.

### 3.9. Drug release studies

Bone cement is commonly used for local antibiotic delivery in the treatment of bone infections.^43^ However, incorporating antibiotics into bone cement can compromise its mechanical strength and often results in suboptimal incomplete drug release. In contrast, bone grafts offer more favorable antibiotic release profiles and mechanical properties compared to antibiotic-loaded bone cement.^44^

However, impregnating bone grafts with aqueous solutions of VAN and GEN leads to a rapid initial drug release, while the sustained release necessary for effective infection control is lacking.^16^ To address this limitation, PLGA has been explored as a delivery vehicle due to its tunable chain architecture, adhesiveness, and biodegradability to facilitate controlled and prolonged antibiotic release, enhancing the efficacy of local antibiotic therapy.^45^

The HIP technique enables the preparation of PLGA particles with hydrophobic drugs, providing a surface without pores, which can reduce the burst release. The release profiles of HIP complexes from PLGA NPs and from allografts impregnated with PLGA/A NPs were investigated.

**Fig. 5:**
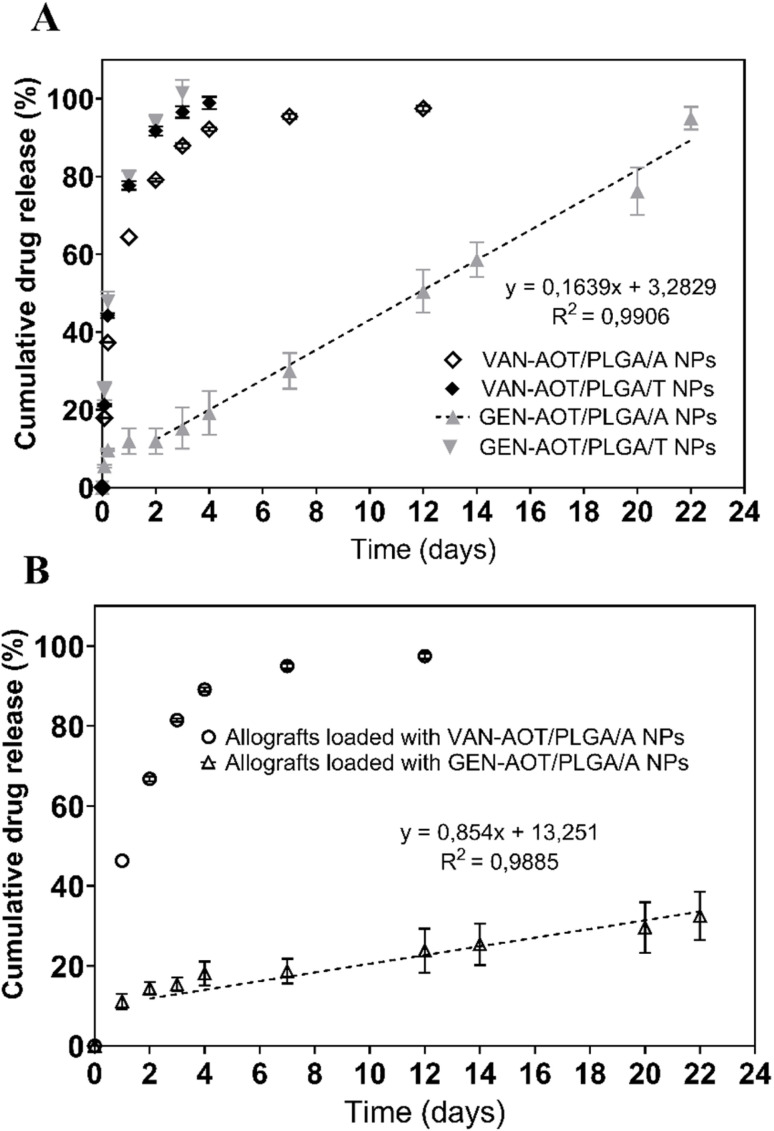
Dissolution profiles of VAN-AOT-loaded PLGA/A nanoparticles (unfilled diamond) and PLGA/T nanoparticles (black diamond), and GEN-AOT-loaded PLGA/A nanoparticles (grey triangle) and PLGA/T nanoparticles (grey inverted triangle) (A); dissolution profiles of VAN-AOT/PLGA/A-loaded allogenic bone grafts (circle) and GEN-AOT/PLGA/A-loaded allogenic bone grafts (unfilled triangle) (B).

As illustrated in Fig. 5A, PLGA/A NPs loaded with GEN-AOT exhibited a low initial burst release of approximately 12%. The GEN-AOT release profile was sustained and linear from day 2 until day 22 (the end of the study), by which point over 90% of the encapsulated drug had been released. In contrast, PLGA/T NPs loaded with GEN-AOT demonstrated a significantly higher burst release, with nearly 80% of the cumulative drug released within the first 24 hours.

A similar release pattern was observed for PLGA/A and PLGA/T NPs loaded with VAN-AOT. Specifically, PLGA/A NPs containing VAN-AOT released approximately 79% of the drug within the first 48 hours. Likewise, PLGA/T NPs released approximately 78% of the drug within the first 24 hours. NPs based on PLGA/A polymer were chosen for further impregnation of allografts.

The dissolution profiles of bone grafts loaded with VAN-AOT/PLGA/A and GEN-AOT/PLGA/A NPs are shown in Fig. 5B. Over 95% of VAN-AOT was released by the end of day 7, maintaining concentrations above 2 µg mL^−1^ throughout this period. Meanwhile, GEN-AOT release from the bone grafts was linear starting from day 2 and reached 33% by day 22, consistently maintaining concentrations above 15 µg mL^−1^ during the entire measurement period.

These results indicate that PLGA-based NPs, particularly when impregnated into bone grafts, can be effectively used for the prolonged local release of antibiotics in the treatment of orthopaedic infections.

## 4. Conclusions

The study suggests the use of bone grafts impregnated with vancomycin and gentamicin loaded PLGA nanoparticles in orthopaedic surgeries for the local treatment of musculoskeletal infections. To overcome the limitations connected with hydrophilic properties of VAN and GEN, the HIP technique was employed using anionic surfactants bis(2-ethylhexyl) sulfosuccinate sodium salt, sodium dodecyl sulfate, sodium dodecylbenzene sulfonate. A novel potentiometric titration method was used to determine the optimal antibiotic-to-surfactant molar ratio, yielding precipitation efficiencies of up to 58% for SDS and up to 80% for the other surfactants. These results confirm the effectiveness of this method in optimizing HIP-complex formation.

For the first time, HIP-complexed VAN was incorporated into PLGA NPs. AOT-based complexes of VAN and GEN were selected as model formulations. NPs prepared using PLGA branched on A demonstrated higher EE for VAN-AOT and GEN-AOT compared to PLGA branched on T. Subsequently, PLGA/A NPs loaded with VAN-AOT and GEN-AOT were utilized to impregnate allogenic bone grafts, with the presence of NPs confirmed on the graft surface. The release profiles obtained from the impregnated grafts suggest their suitability for sustained antibiotic delivery in the context of infection management. Antibacterial action of prepared NPs was preserved or slightly improved confirming their suitability in MSIs management. These results highlight the promising potential of these NPs for clinical application in bone graft impregnation and localized antibiotic therapy.

## Ethical statement

The bone grafts were manipulated by a fully licensed tissue bank (registration number MTB 06, European Union Tissue Establishment Code CZ 000427), and the research was approved by the Local Ethics Committee (Reference Number 201107 S08P, 07/2011).

## Author contributions

Vladislav Frolov: conceptualization, methodology, investigation, writing – original draft, writing – review & editing, visualization. Tamás Sovány: conceptualization, methodology, writing – review & editing, visualization, supervision. Jan Loskot: methodology, investigation, writing – review & editing, visualization, supervision. Edit Csapó: conceptualization, methodology, supervision. Norbert Varga: conceptualization, methodology, supervision. Alharith A. A. Hassan: methodology, investigation, writing – review & editing. Aleš Bezrouk: methodology, investigation, writing – review & editing. Klára Konečná: methodology, investigation, writing – review & editing. Ondřej Janďourek: methodology, investigation, writing – review & editing. Eva Šnejdrová: conceptualization, methodology, writing – review & editing, visualization, supervision.

## Conflicts of interest

The authors declare that they have no known competing financial interests or personal relationships that could have appeared to influence the work reported in this paper.

## Supplementary Material

RA-015-D5RA04263A-s001

RA-015-D5RA04263A-s002

RA-015-D5RA04263A-s003

RA-015-D5RA04263A-s004

RA-015-D5RA04263A-s005

RA-015-D5RA04263A-s006

RA-015-D5RA04263A-s007

RA-015-D5RA04263A-s008

RA-015-D5RA04263A-s009

RA-015-D5RA04263A-s010

RA-015-D5RA04263A-s011

## Data Availability

All data supporting this study are available within the paper and its supplementary information (SI). Supplementary information is available. See DOI: https://doi.org/10.1039/d5ra04263a.
