# Supplementary figures and images for: Ion-paired antibiotics in PLGA nanoparticles: improving encapsulation efficiency and musculoskeletal infection treatment
Source: RSC Adv. 2025 Nov 24;15(54):46024–36. doi: 10.1039/d5ra04263a (PMC12643545; doi:10.1039/d5ra04263a)

Electron Image 89

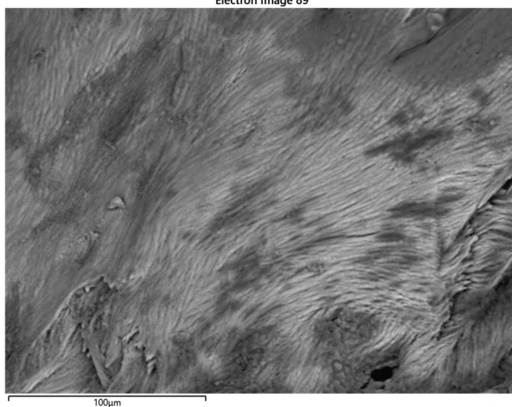

P K $\alpha$ 1

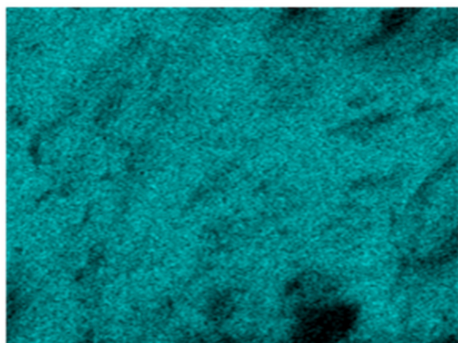

Ca K $\alpha$ 1

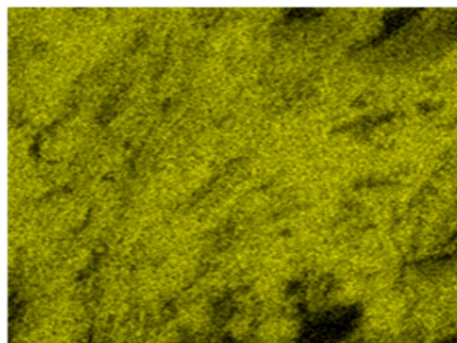

C K $\alpha$ 1,2

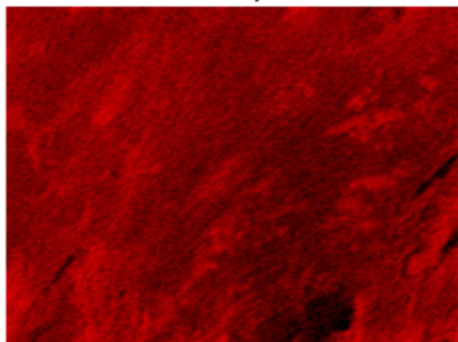

O K $\alpha$ 1

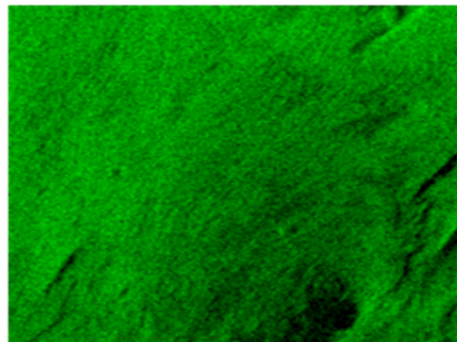

S K $\alpha$ 1

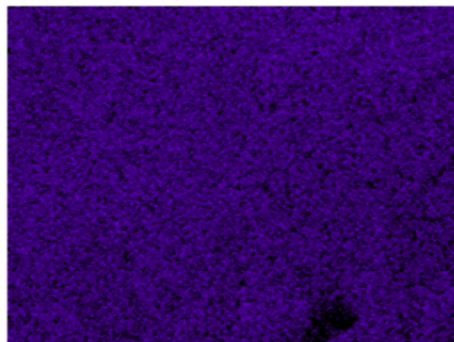

Supplement: RA-015-D5RA04263A-s002 [file RA-015-D5RA04263A-s002.pdf]

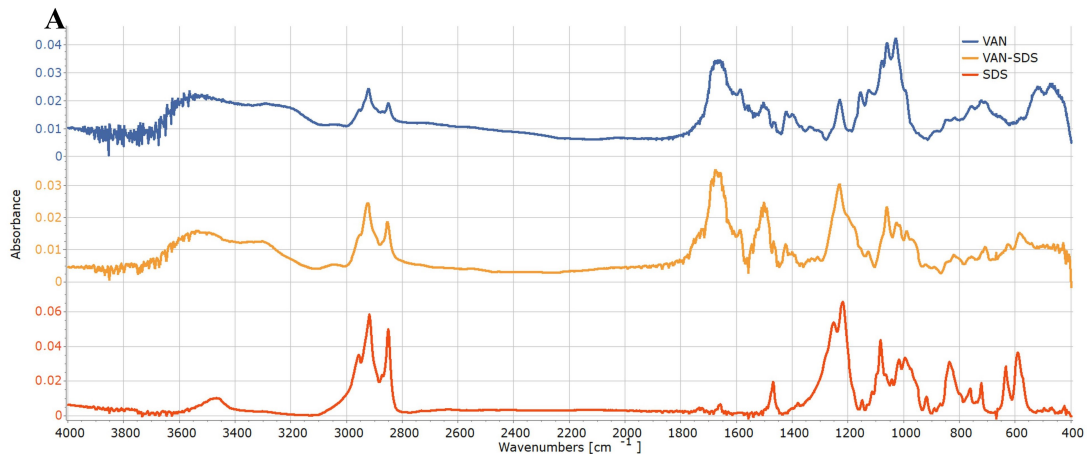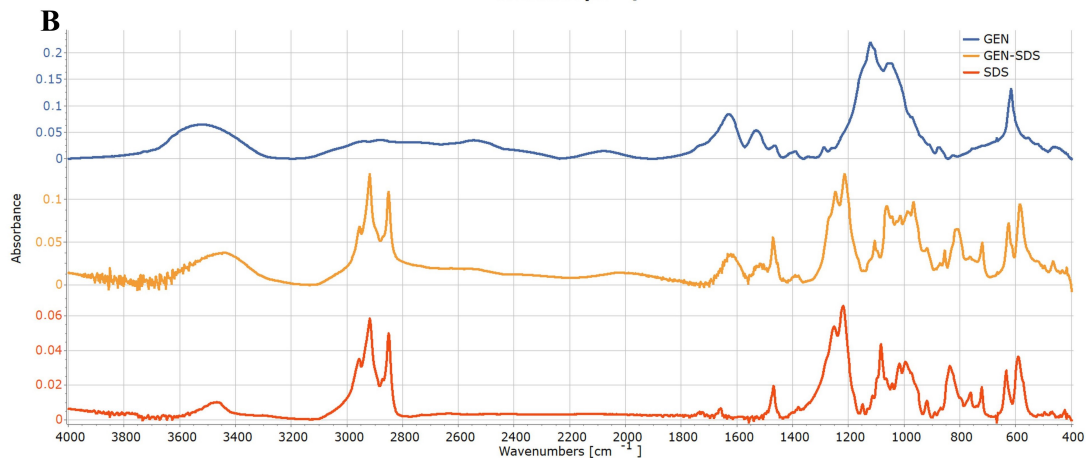

Supplement: RA-015-D5RA04263A-s004 [file RA-015-D5RA04263A-s004.pdf]

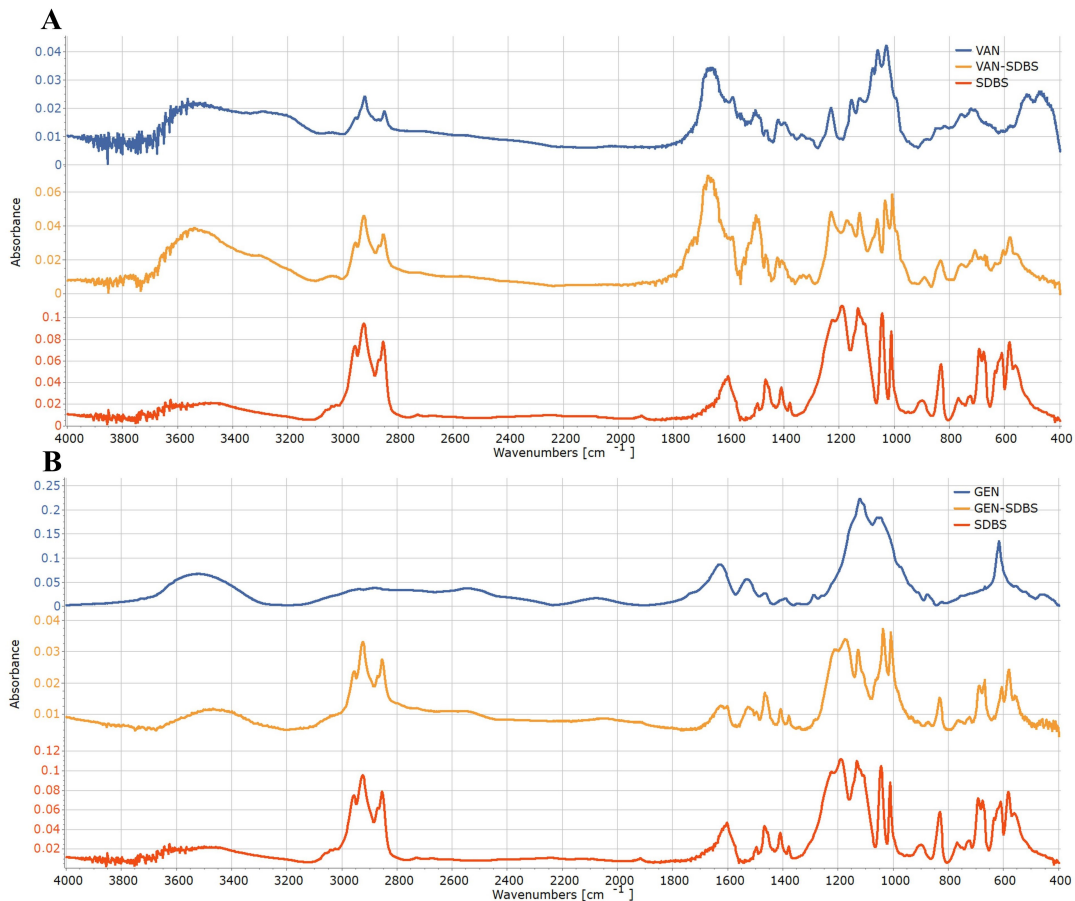

Supplement: RA-015-D5RA04263A-s005 [file RA-015-D5RA04263A-s005.pdf]

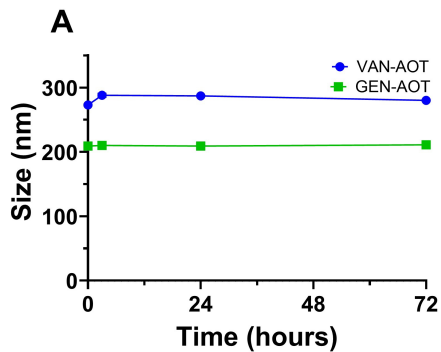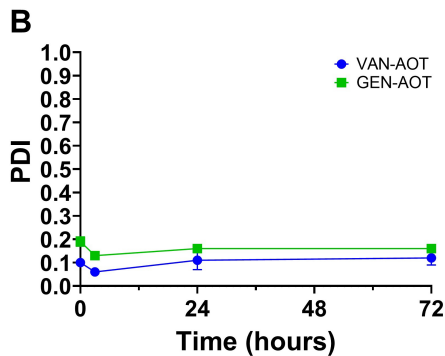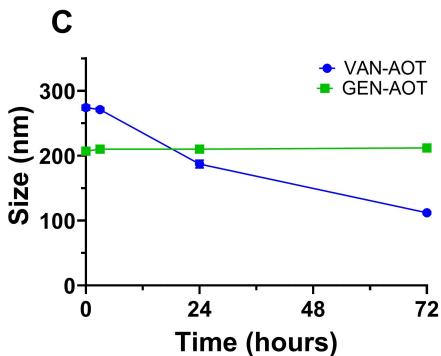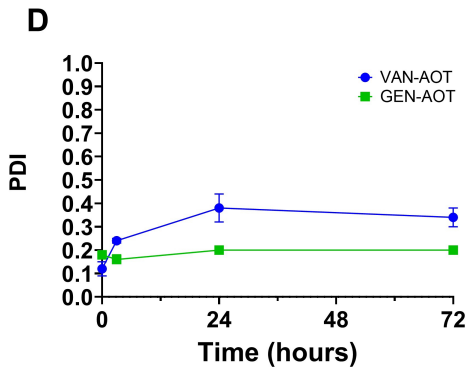

Supplement: RA-015-D5RA04263A-s006 [file RA-015-D5RA04263A-s006.pdf]

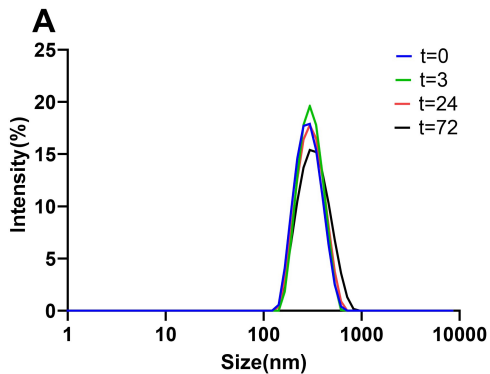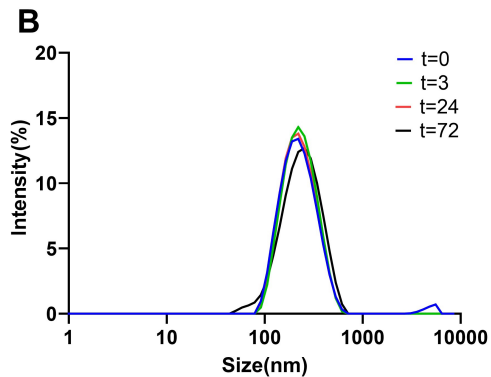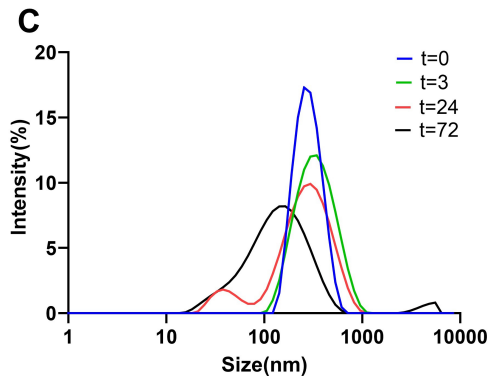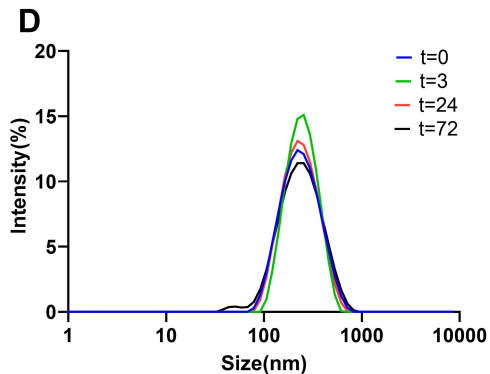

Supplement: RA-015-D5RA04263A-s007 [file RA-015-D5RA04263A-s007.pdf]

Electron Image 54

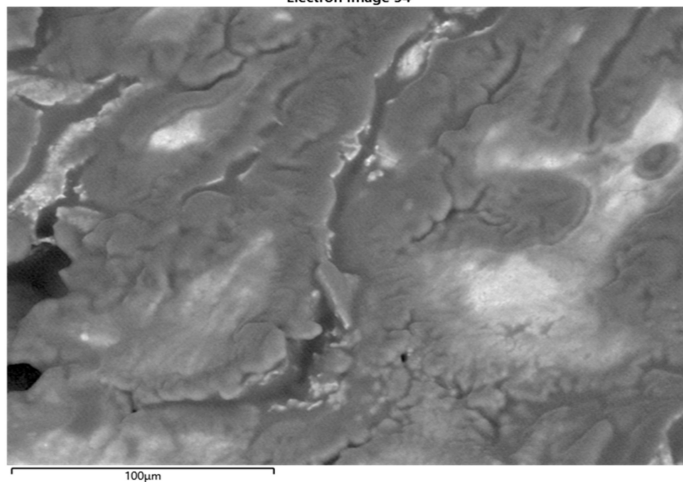

P K $\alpha$ 1

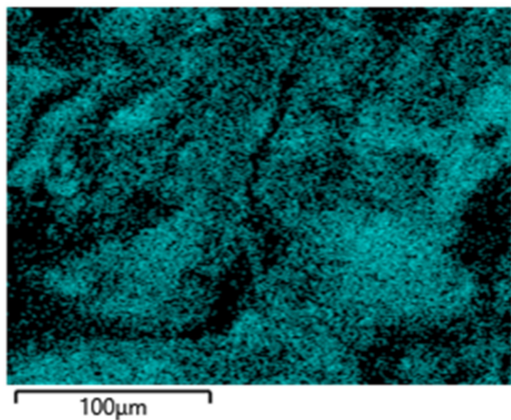

C K $\alpha$ 1,2

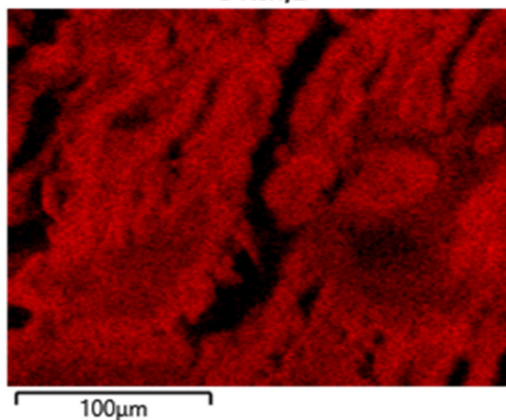

Ca K $\alpha$ 1

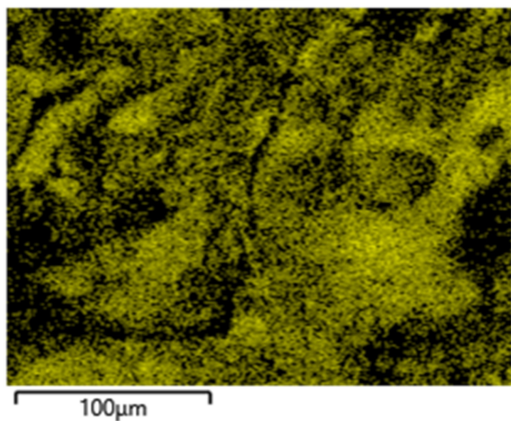

O K $\alpha$ 1

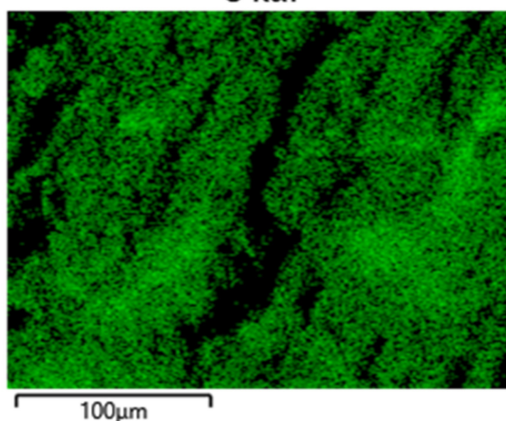

Supplement: RA-015-D5RA04263A-s008 [file RA-015-D5RA04263A-s008.pdf]

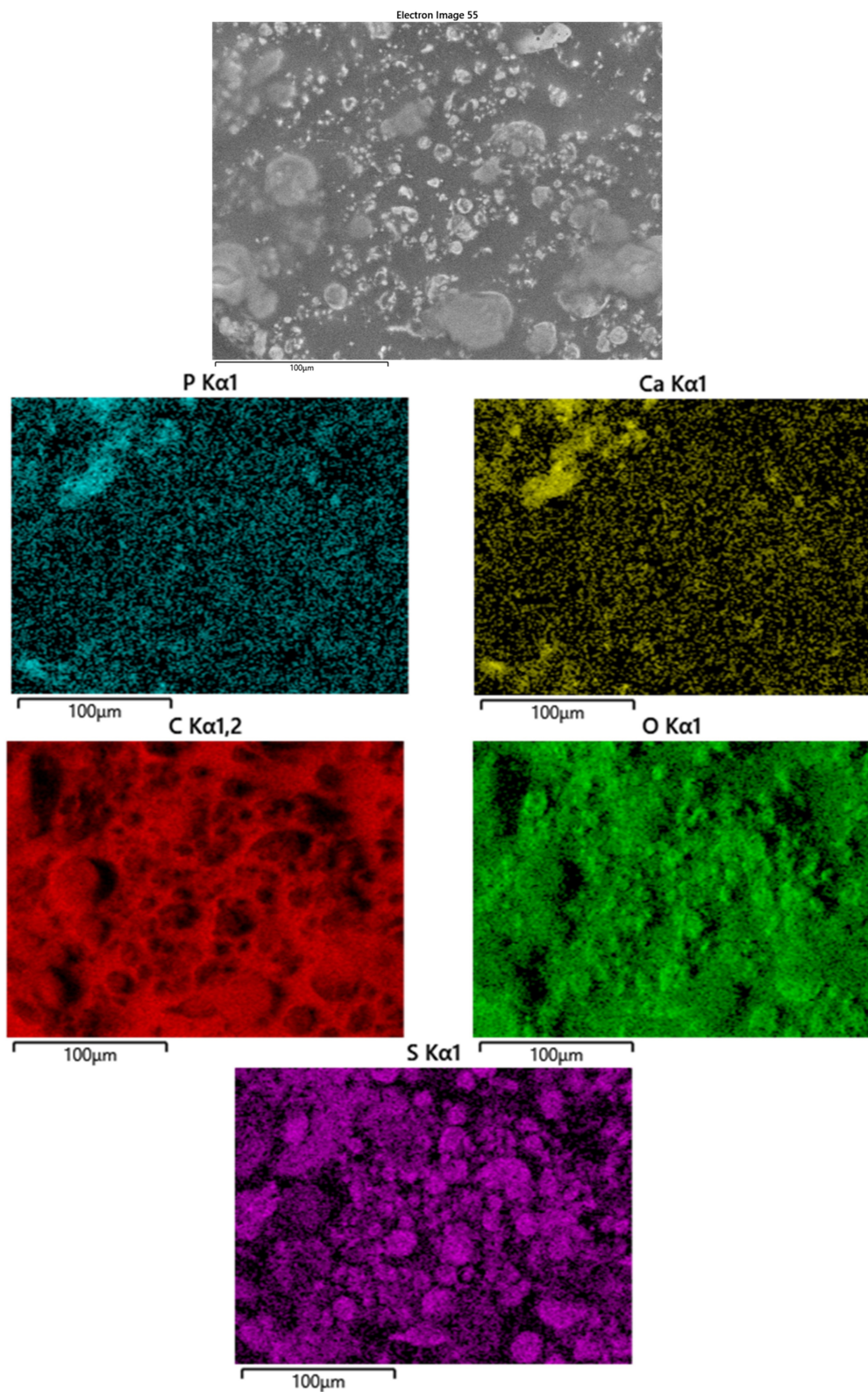

Supplement: RA-015-D5RA04263A-s009 [file RA-015-D5RA04263A-s009.pdf]

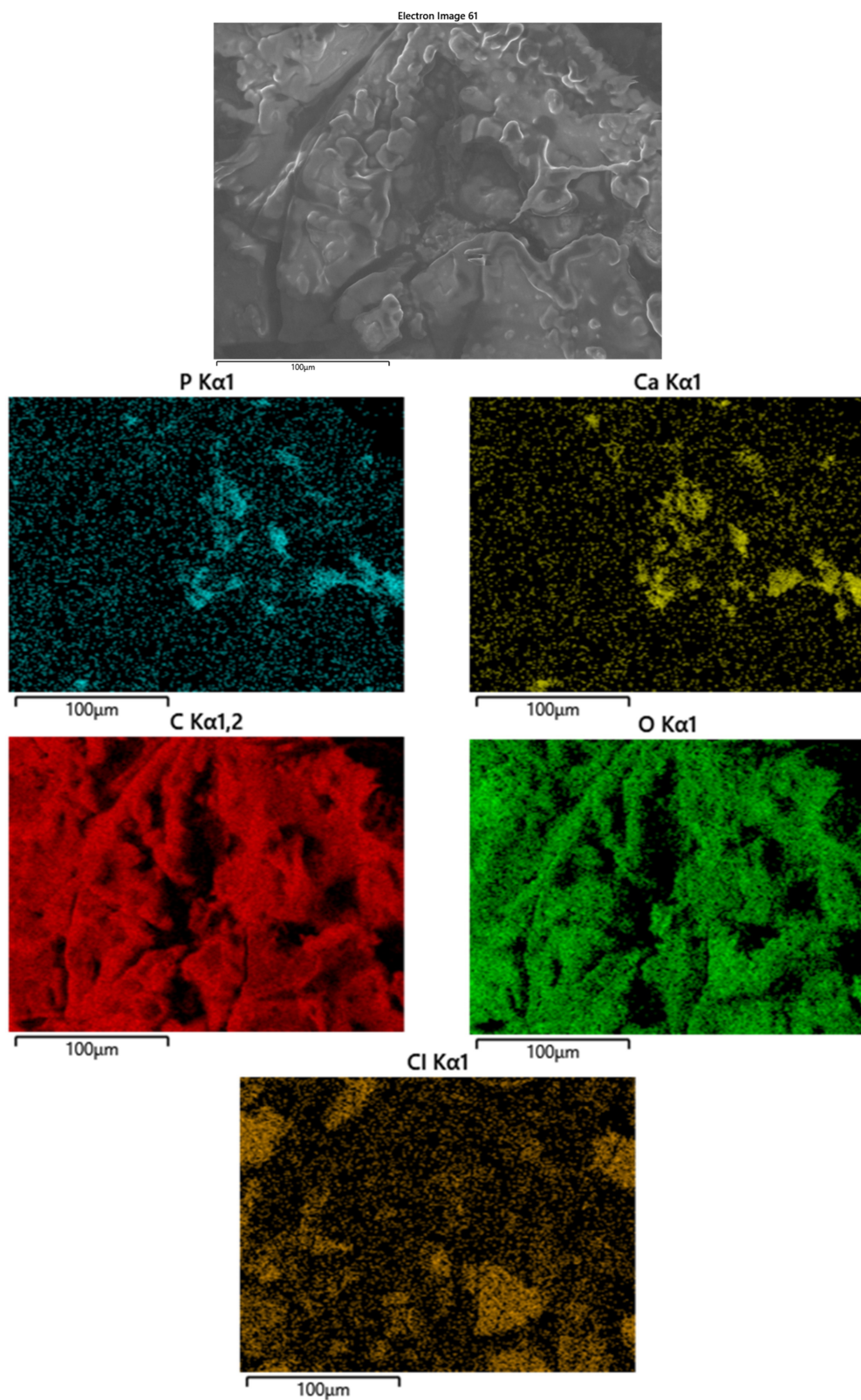

Supplement: RA-015-D5RA04263A-s010 [file RA-015-D5RA04263A-s010.pdf]

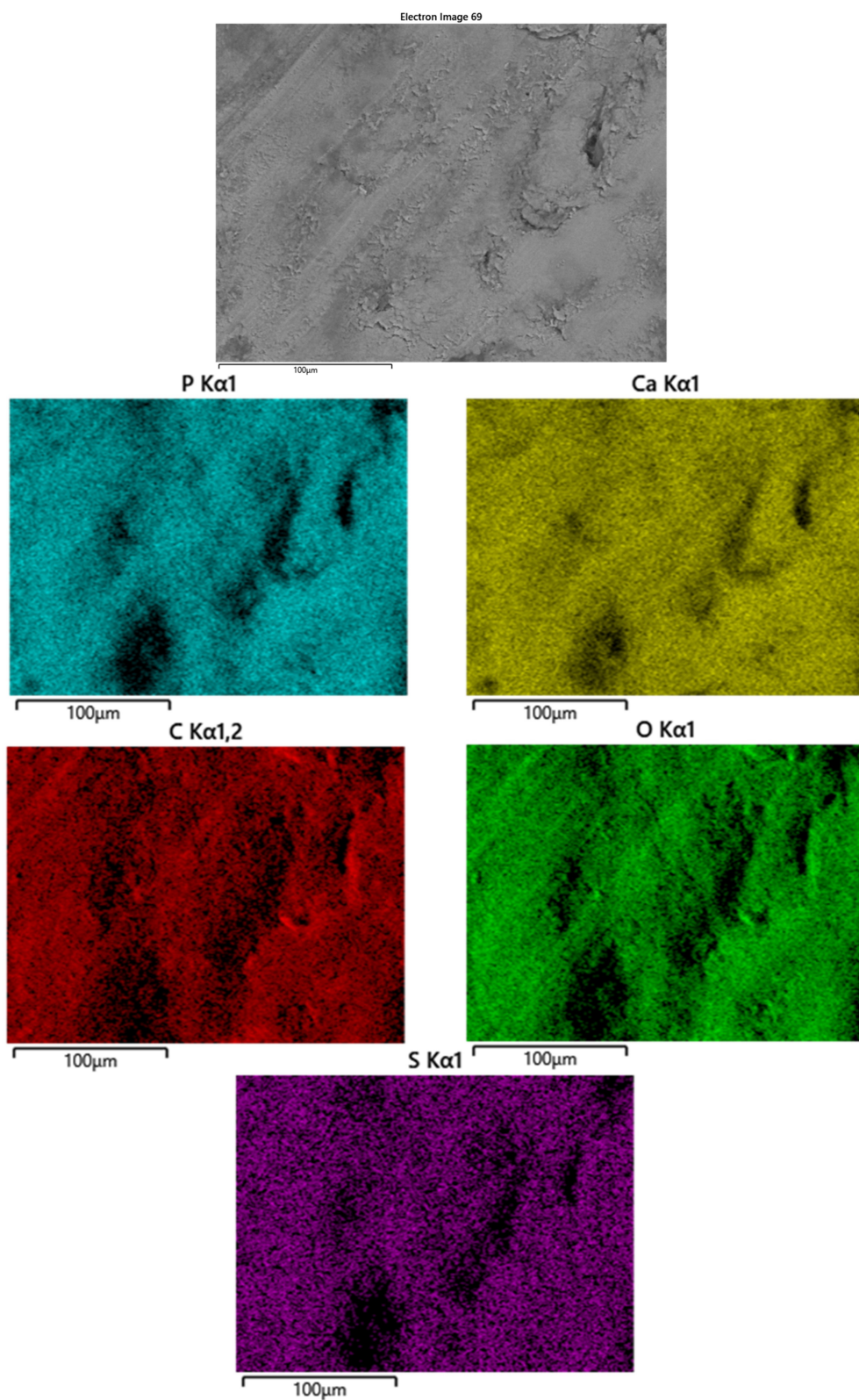

Supplement: RA-015-D5RA04263A-s011 [file RA-015-D5RA04263A-s011.pdf]
