# Supplementary material for: Ion-paired antibiotics in PLGA nanoparticles: improving encapsulation efficiency and musculoskeletal infection treatment
Source: RSC Adv. 2025 Nov 24;15(54):46024–36. doi: 10.1039/d5ra04263a (PMC12643545; doi:10.1039/d5ra04263a)
Supplement: RA-015-D5RA04263A-s003 [file RA-015-D5RA04263A-s003.pdf]

Supplementary Information (SI) for RSC Advances.
This journal is © The Royal Society of Chemistry 2025

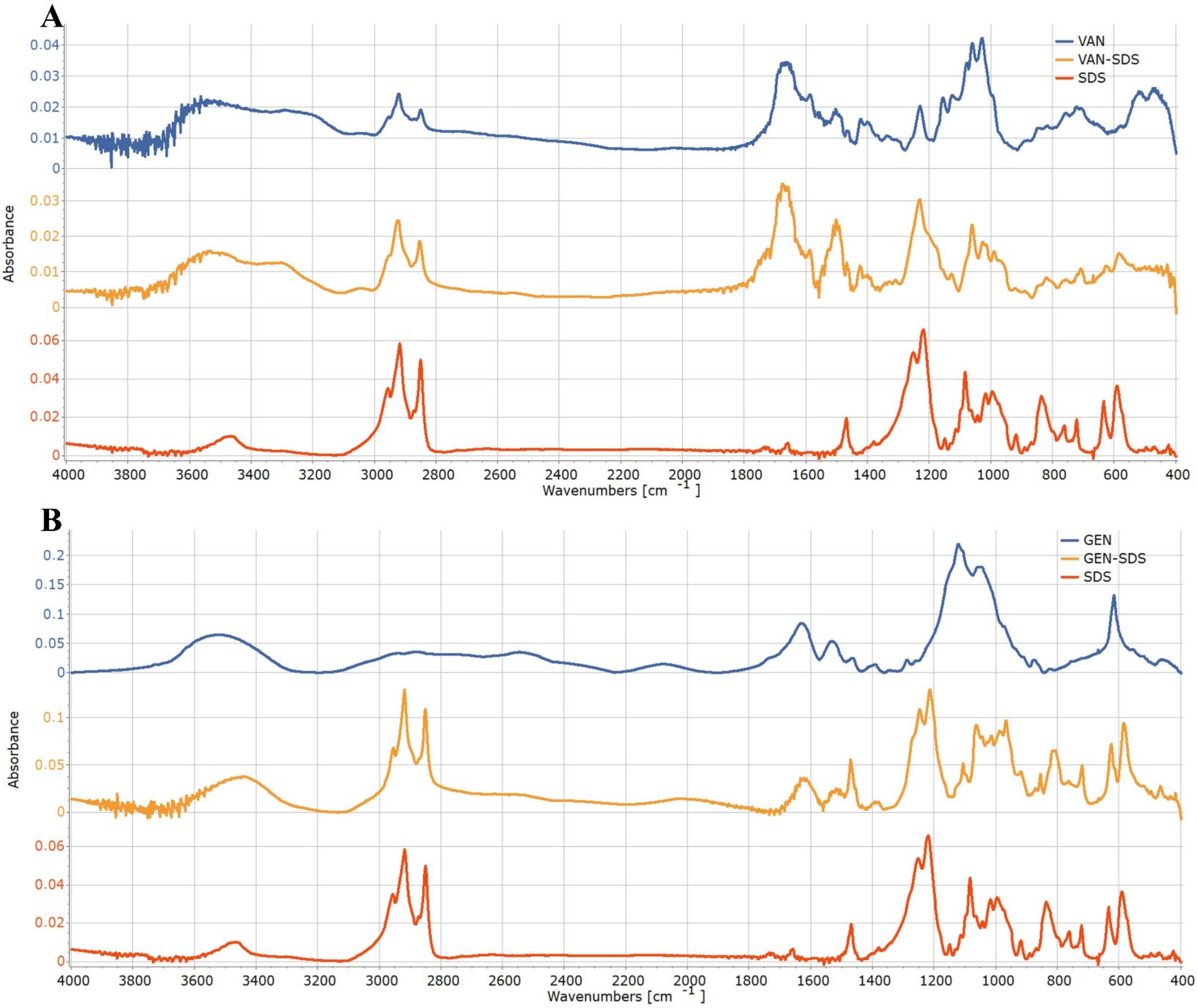

Supplementary Materials

Fig. S1. FTIR spectra of vancomycin (VAN) (A) and gentamicin (GEN) (B) complexes with sodium dodecyl sulfate (SDS). SDS (red line), vancomycin or gentamicin (blue line) and the respective complex with SDS (orange line).

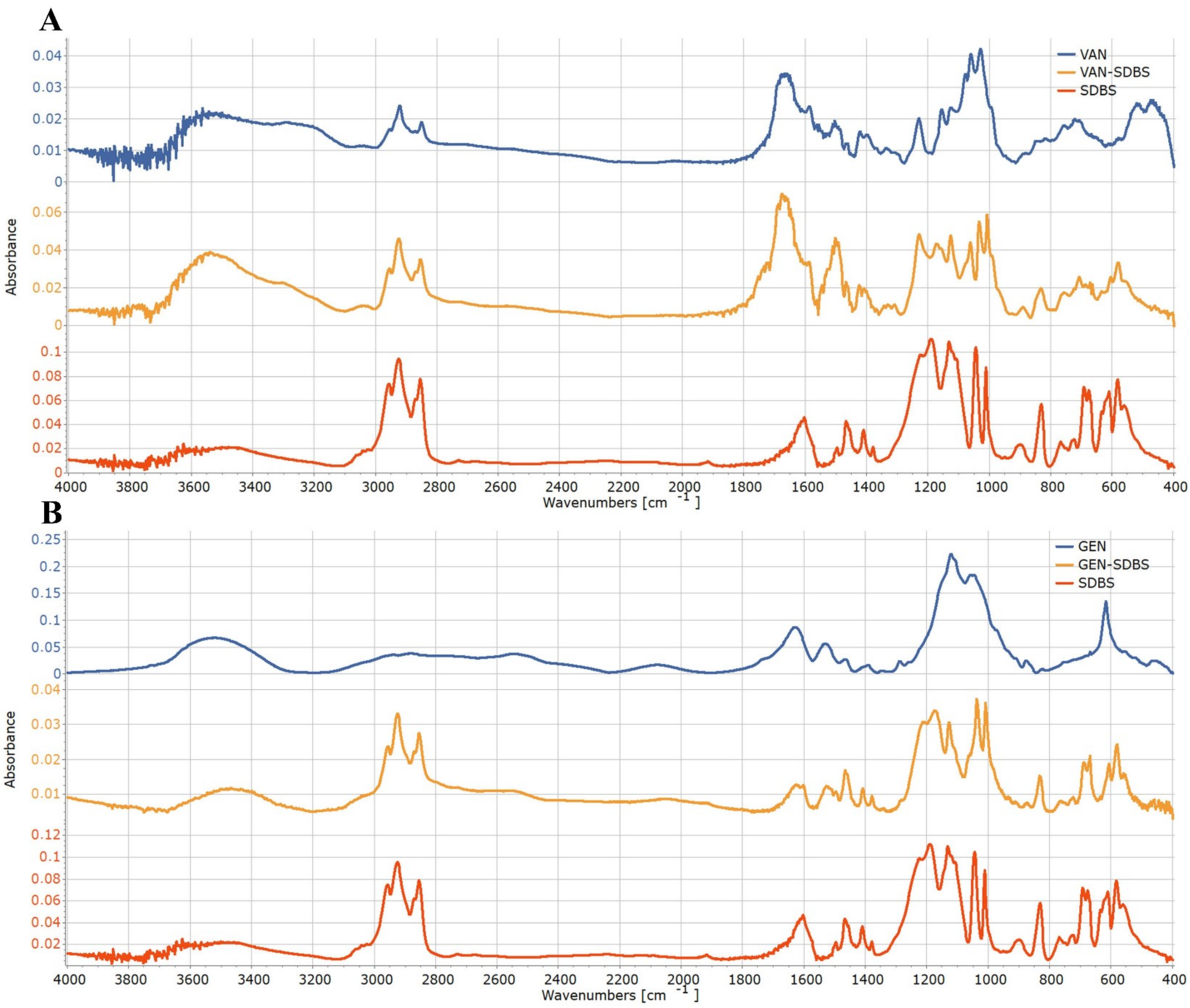

Fig. S2. FTIR spectra of vancomycin (VAN) (A) and gentamicin (GEN) (B) complexes with sodium dodecyl sulfate (SDBS). SDBS (red line), vancomycin or gentamicin (blue line) and the respective complex with SDBS (orange line).

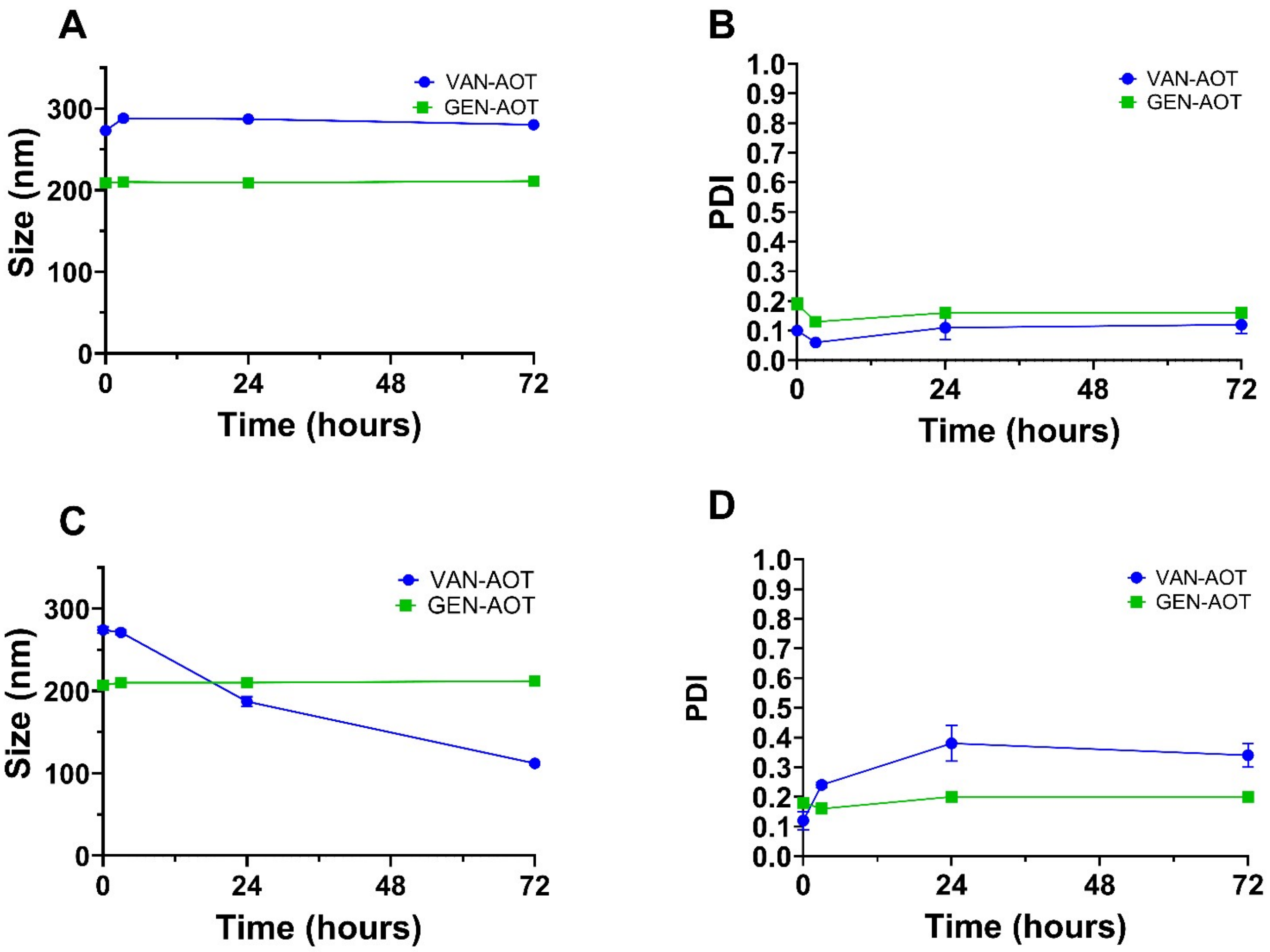

Fig. S3. Stability of PLGA/A nanoparticles upon dilution in aqueous media over time. Average particle size (A) and polydispersity index (B) of PLGA/A nanoparticles loaded with vancomycin or gentamicin hydrophobic ion pair with AOT in Milli-Q water, and average particle size (C) and polydispersity index (D) of the same nanoparticles diluted in PBS.

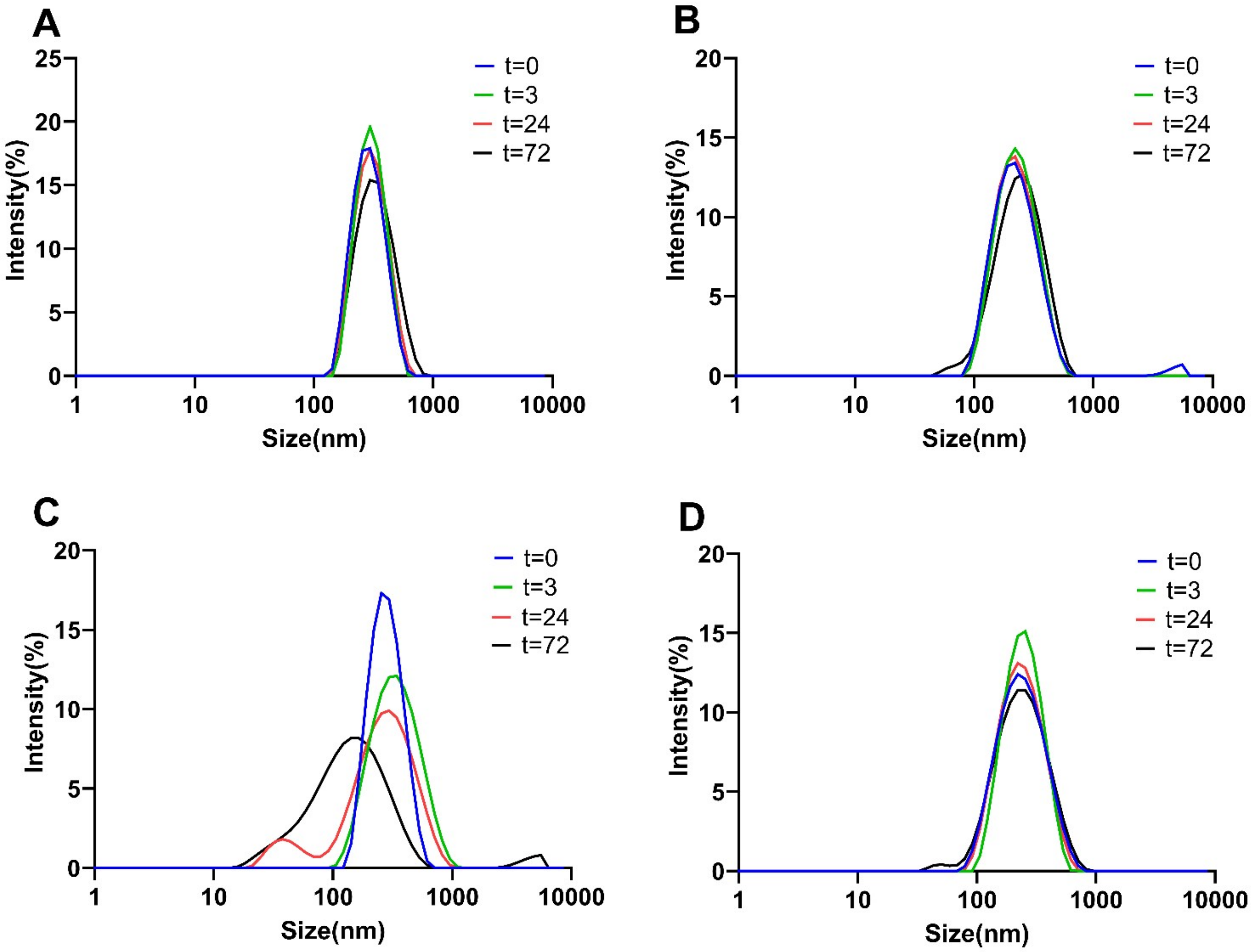

Fig. S4. Size distributions of PLGA/A nanoparticles loaded with the hydrophobic ion pair of vancomycin (A) or gentamicin (B) with AOT after dilution in Milli-Q water, and PLGA/A nanoparticles loaded with the hydrophobic ion pair of vancomycin (C) or gentamicin (D) with AOT after dilution in PBS.

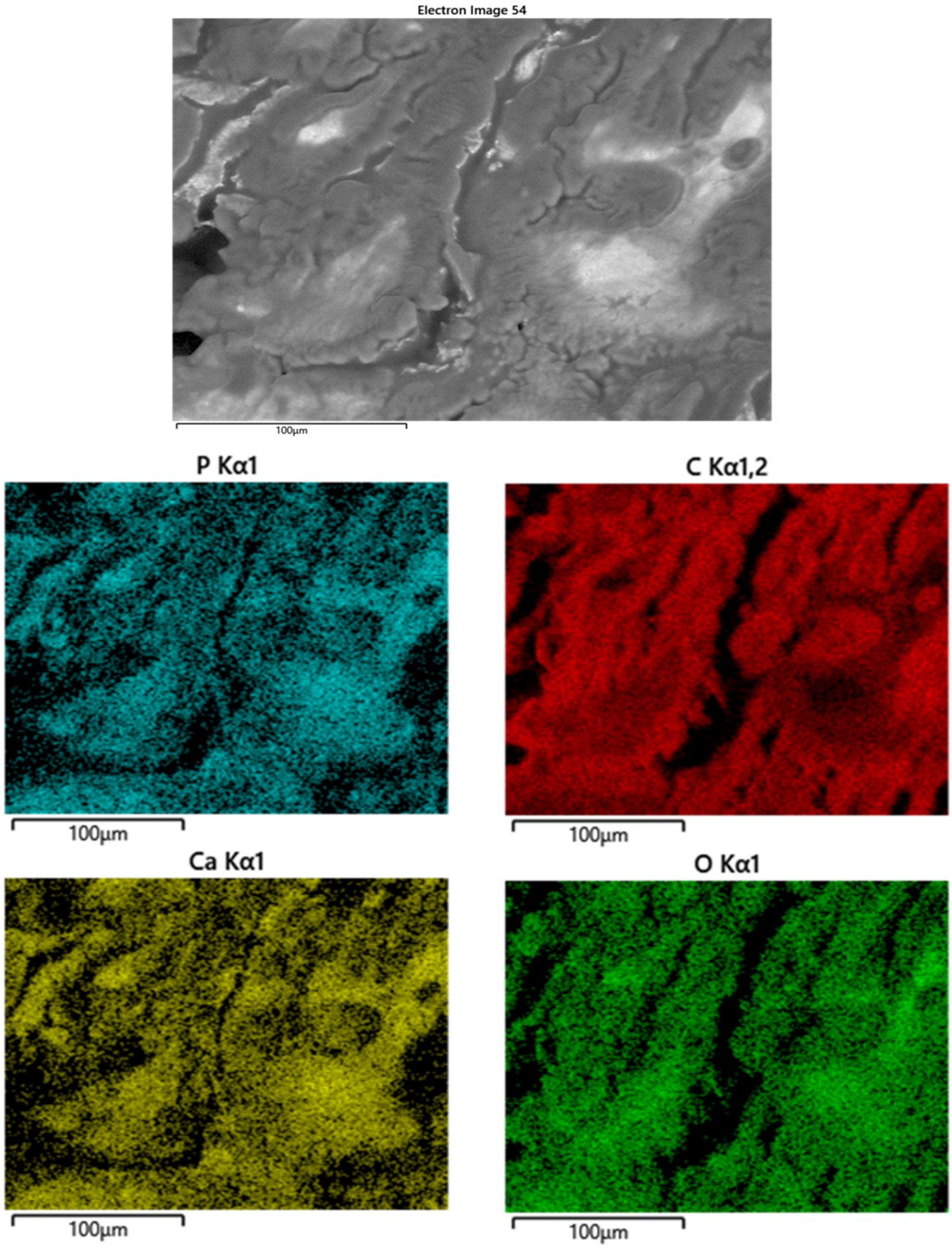

Fig. S5. EDS mapping of blank allogenic bone graft.

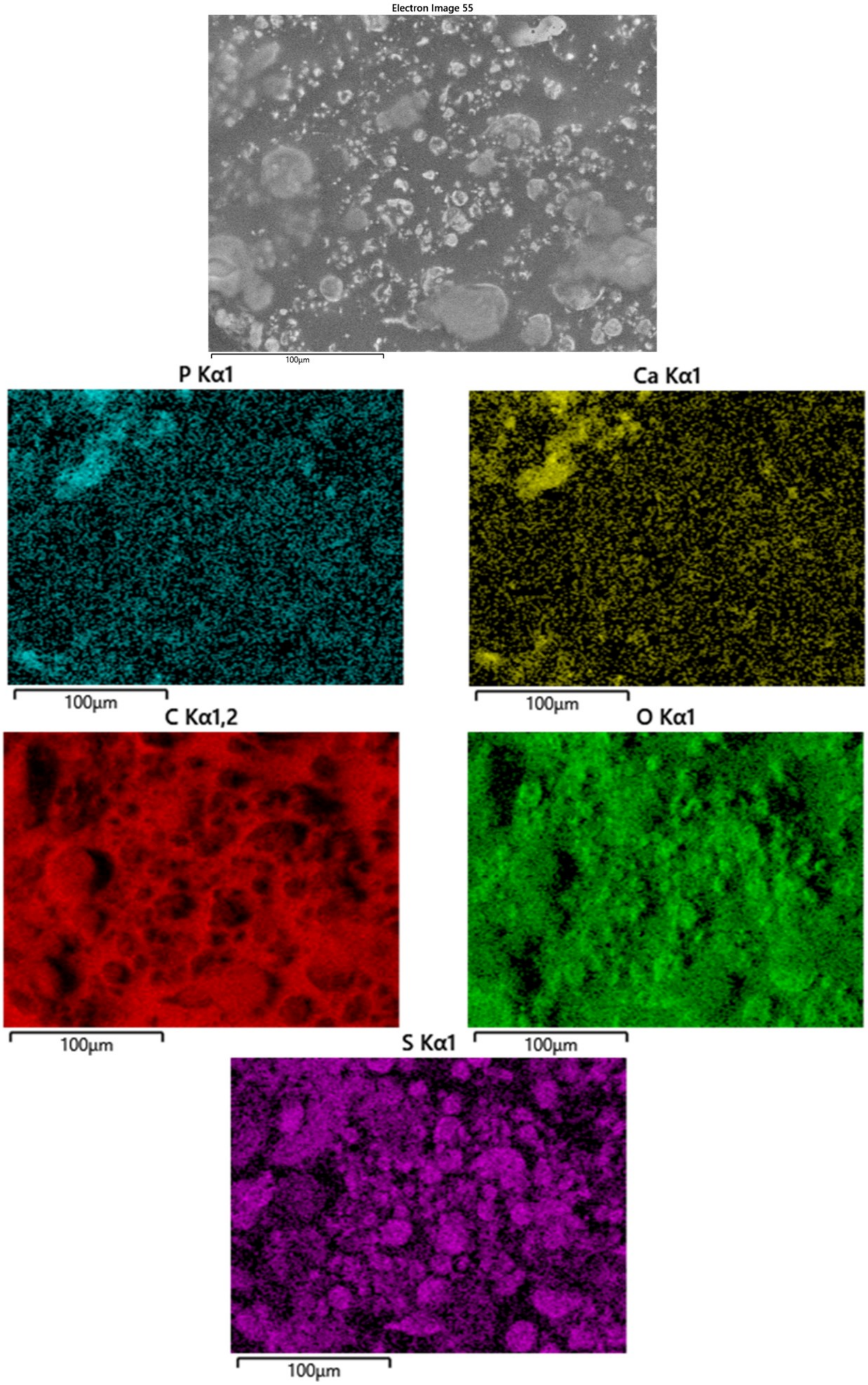

Fig. S6. EDS mapping of allogenic bone graft loaded with the gentamicin sulfate substance

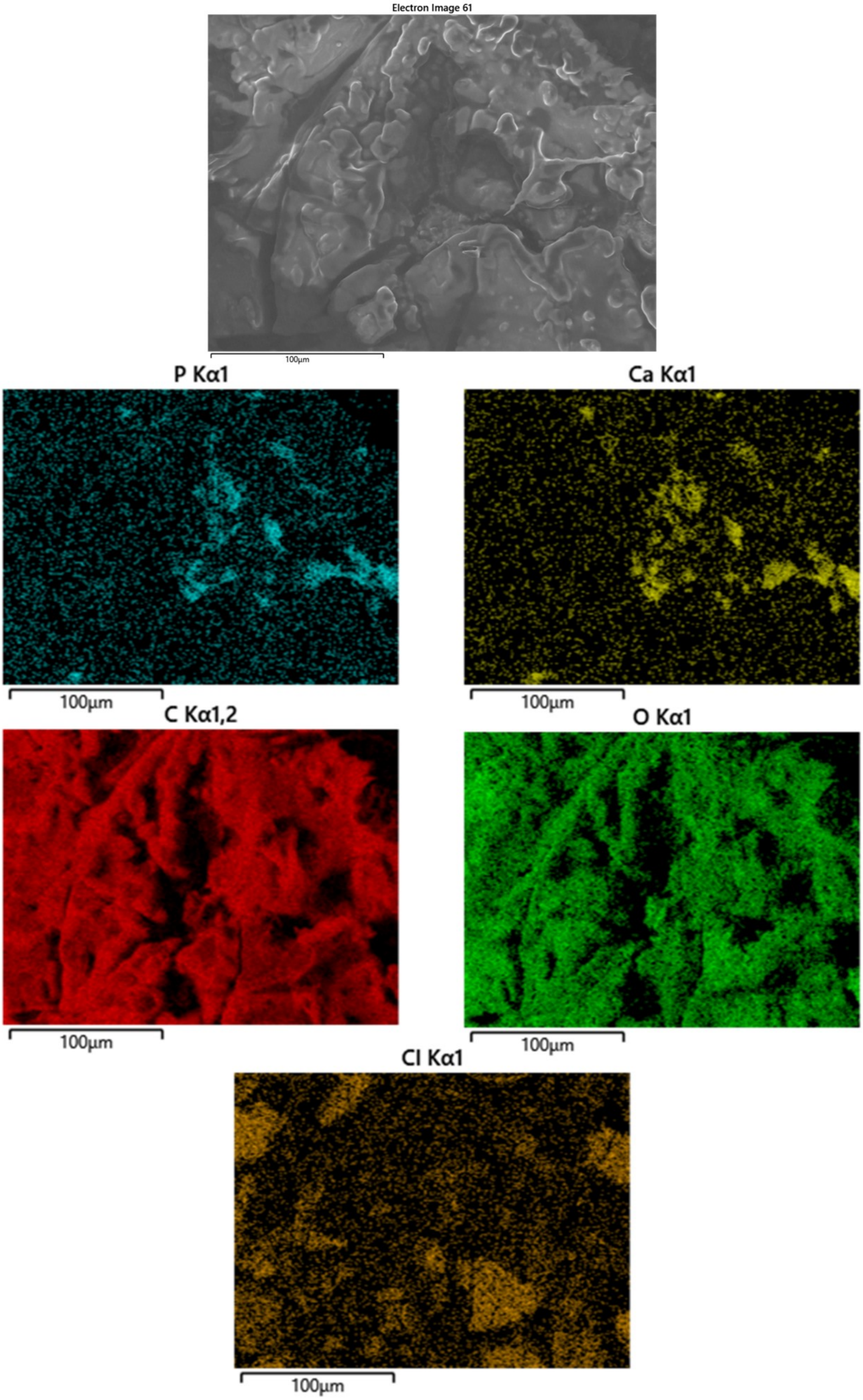

Fig. S7. EDS mapping of allogenic bone graft loaded with the vancomycin hydrochloride substance

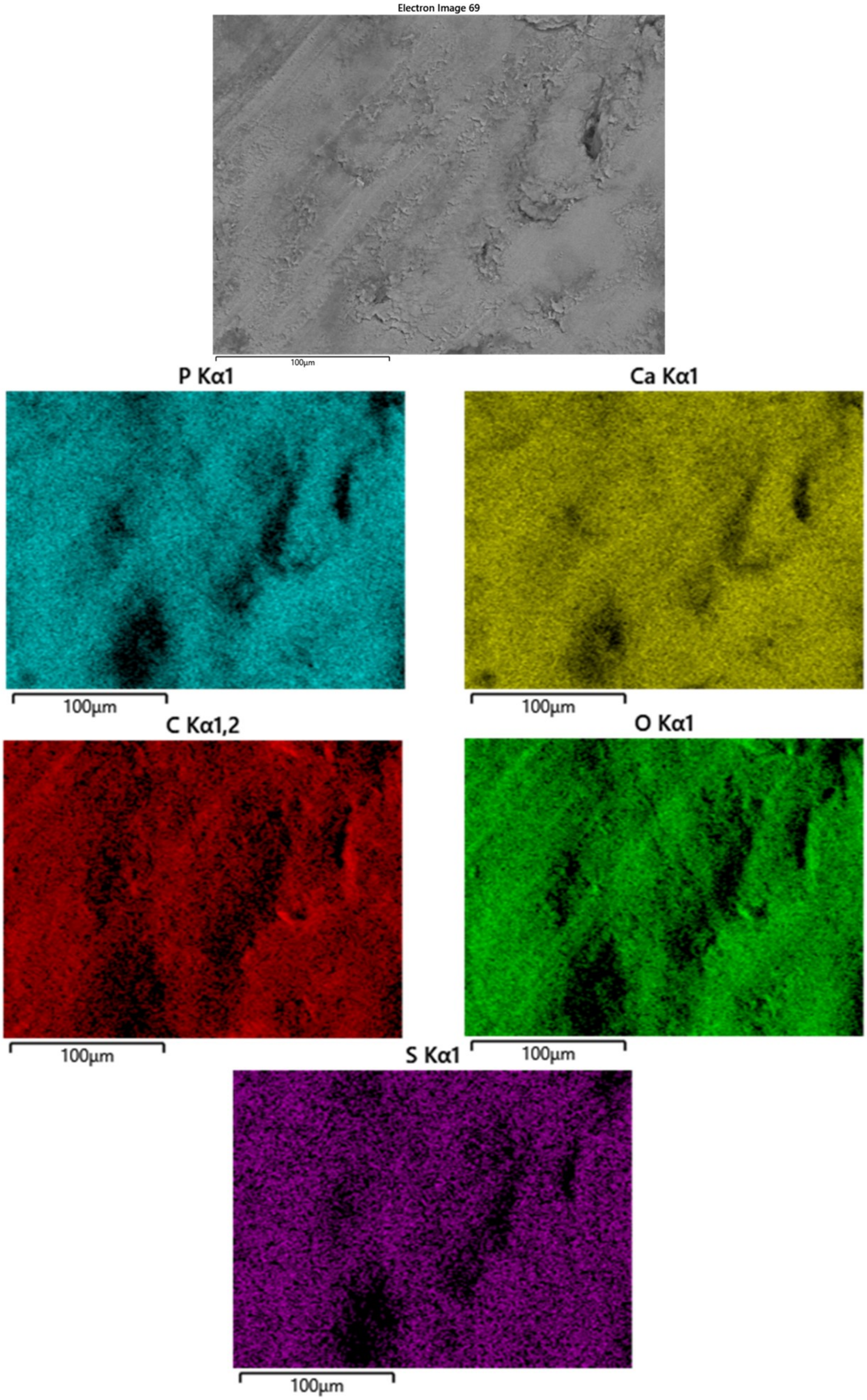

Fig. S8. EDS mapping of allogenic bone graft loaded with the PLGA/A nanoparticles with the gentamicin hydrophobic ion pair with AOT

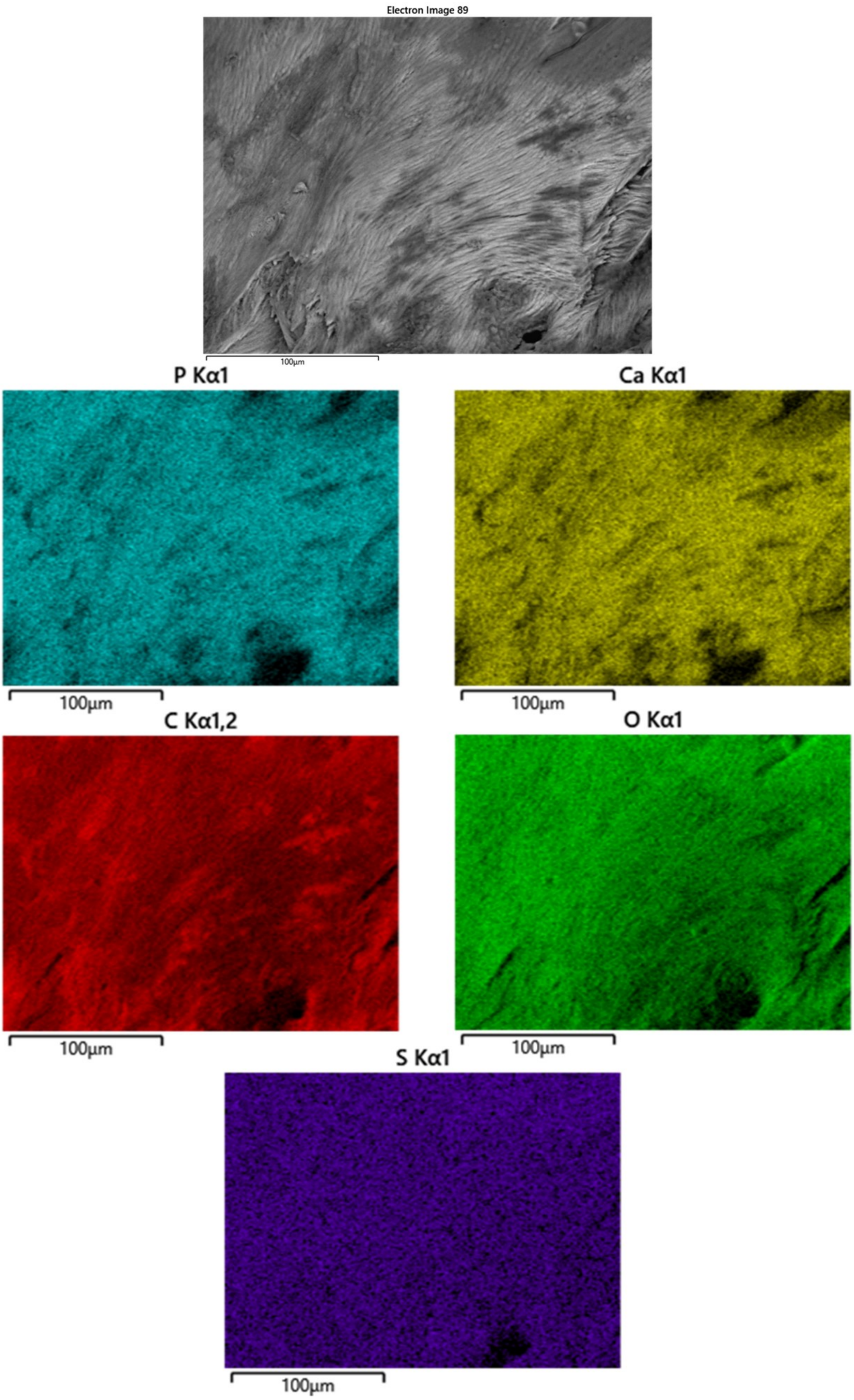

Fig. S9. EDS mapping of allogenic bone graft loaded with the PLGA/A nanoparticles with the vancomycin hydrophobic ion pair with AOT
